# Trend analysis of hematological tumors in adolescents and young adults from 1990 to 2019 and predictive trends from 2020 to 2044: A Global Burden of Disease study

**DOI:** 10.1002/cam4.70224

**Published:** 2024-10-03

**Authors:** Linlin Huang, Jingsong He

**Affiliations:** ^1^ Department of Hematology, Bone Marrow Transplantation Center, School of Medicine, The First Affiliated Hospital Zhejiang University Hangzhou Zhejiang China; ^2^ Department of Hematology, The First Affiliated Hospital of Zhejiang Chinese Medical University (Zhejiang Provincial Hospital of Chinese Medicine) Hangzhou Zhejiang China

**Keywords:** epidemiology, hematological malignancies, leukemia, lymphoma

## Abstract

**Introduction:**

Cancer constitutes the primary disease spectrum contributing to the Global Burden of Disease (GBD). Adolescents and young adults (AYA) aged 15–39 have received relatively less attention regarding tumor prevention, diagnosis, and treatment compared to older adults and children. This study aimed to analyze the changes in the disease burden of hematological malignancies among the global AYA over the past three decades based on the GBD database.

**Methods:**

The changes in the disease burden of hematological malignancies were analyzed among the AYA over the past three decades based on the information from the GBD database. The future trends were predicted using the Nordpred package in R.

**Results:**

Our results showed that leukemia ranked first as the leading tumor burden among AYA in 2019, but the incidence rate and mortality rate of leukemia decreased year by year, with a projected age‐standardized incidence rate (ASIR) of 1.65/100,000 for females and 2.40/100,000 for males by the year 2044. In addition, the incidence of non‐Hodgkin‘s lymphoma has been gradually increasing in recent years, with an ASIR of 1.73/100,000 from 2020 to 2024. The results may serve as a basis for developing strategies to reduce the burden of hematological malignancies in the AYA population in different regions.

## 1 INTRODUCTION

Cancer constitutes the primary disease spectrum contributing to the Global Burden of Disease (GBD), ranking second only to cardiovascular diseases.^1^, ^2^ Furthermore, predictions suggest that the disease burden caused by malignant tumors will continue to increase in the future.^1^, ^2^ Adolescents and young adults (AYA) aged 15–39 are the backbone of societal production and development and have received relatively less attention regarding tumor prevention, diagnosis, and treatment compared with older adults and children.^3^, ^4^, ^5^, ^6^ In addition, the incidence patterns of cancers differ in this age group compared with older adults and children, with hematological malignancies being a significant component of tumor occurrence among the AYA.^3^, ^7^ Lymphomas and leukemias are the most common hematological cancers in AYAs.^8^ The Surveillance, Epidemiology, and End Results (SEER) database showed that lymphomas and leukemias ranked 4th and 10th of all cancers in female AYAs (incidence of 6.9 per 100,000 and 2.7 per 100,000, respectively) while ranked 2nd and 8th in male AYAs (incidence of 7.7 and 2.8 per 100,000, respectively).^8^ In the United States of America, leukemia represented the second and fifth most common causes of cancer‐related deaths in AYAs in 2015, while those cancers represented the second and third most common causes of cancer death in male AYAs, and the third and fifth in female AYAs in Latin America in 1998–2007.^9^, ^10^

Recently, with the advancements in the fields of hematology and oncology, there have been significant improvements in the diagnosis and treatment of hematological cancers, leading to notable changes in detection rates and survival rates for patients.^11^, ^12^ Therefore, it is crucial to investigate the global burden of hematological malignancies in the 15–39 age group, aiming to provide more information to control and prevent subsequent diseases and alleviate the societal burden. The AYA population features specific medical, social, and psychological characteristics that, when combined with the specific features of hematological malignancies in this population, result in a complex challenge in management; unfortunately, such cancers remain under‐studied in AYAs.^13^ Studying the precise characteristics of hematological cancers in AYAs should help improve diagnosis, treatment, and outcomes.

The GBD database provides comprehensive estimates of the incidence, deaths, and prevalence of diseases for each country and territory. It leverages a wide array of data sources including national and international health surveys, hospital records and clinical research, death records and registries. Studies based on the GBD database have been widely reported.^14^, ^15^

Therefore, this study aimed to analyze the changes in the disease burden of hematological malignancies, including leukemia, Hodgkin's lymphoma, non‐Hodgkin's lymphoma, and multiple myeloma, among the global population aged 15–39 over the past three decades based on the information from the GBD database. The results could help the prevention and management of hematological cancers in AYA.

## 2 MATERIALS AND METHODS

### 2.1 Data source

This study data was derived from the GBD study conducted in 2019. The study encompassed data from 1990 to 2019 and provided comprehensive insights into health outcomes and risks, including mortality rates and disability, across different countries, time periods, ages, and sexes.^2^, ^3^, ^16^ It quantified the health burden caused by 369 diseases, injuries, and risk factors, allowing to witness how disease patterns evolved over time and gain insights into regional disparities (https://vizhub.healthdata.org/gbd‐results/).

The population forecast data used in the present study was sourced from the 2019 revised edition of the World Population Outlook (https://population.un.org/wpp/Download/Standard/Population/). The standard population data comes from the National Cancer Institute (https://seer.cancer.gov/stdpopulations/world.who.html).

### 2.2 Definitions

The term “adolescent and young adults” (AYA) used in this study refers to individuals aged 15–39 years, as defined by the US National Cancer Institute, the AYA Working Group of the European Society for Medical Oncology, and the European Society for Pediatric Oncology.^3^, ^4^

The socio‐demographic index (SDI) is a composite measure used to assess socio‐demographic development in different regions or countries. It considers factors such as per capita income, educational attainment, and fertility rate to provide a comprehensive summary of socio‐demographic development. In this study, countries were ranked and categorized into five equal SDI groups (low, low‐middle, middle, high‐middle, and high SDI) to examine variations in disease burden across different resource contexts.

The 204 countries and territories found in GBD were divided into 21 regions, including Central Asia, Central Europe, Eastern Europe, High‐income Australia, Asia Pacific, North America, Southern Latin America, Western Europe, Andean Latin America, Caribbean, Central Latin America, Tropical Latin America, North Africa, Middle East, South Asia, East Asia, Oceania, Southeast Asia, and Central, Eastern, Southern, and Western Sub‐Saharan Africa.

The age‐standardized rates were the age‐standardized incidence rate (ASIR) and age‐standardized death rate (ASDR), calculated by adjusting for age distribution using the “epitools” package. All rates were reported per 100,000 person‐years.

### 2.3 Models

The estimated annual percentage change (EAPC) was calculated based on the ASIRs and ASDRs to capture the trends in cancer burden. The calculation used a regression model fitted to the natural logarithm of the rate, represented as ln(rate) = *α* + *β* × (calendar year) + *ε*. EAPC was defined as 100 × (exp(*β*) − 1), and the 95% uncertainty interval (UI) of the EAPC was determined using the fitted model. A comparison was made between the data from 2019 and 1990 to ascertain variations in each outcome.

In order to project the ASIR and ASDR from 2020 to 2044, a log‐linear age period‐cohort model incorporating exponential growth leveling and limited linear trend projection was fitted to recent trends. This model was implemented using the “Nordpred” package in the R software, known to demonstrate empirical effectiveness in projecting current cancer incidence trends into the future.^17^, ^18^ The data were aggregated in 5‐year intervals, and a subgroup analysis was performed specifically for individuals aged 15–39 years.

### 2.4 Statistical analysis

The data analyses were conducted using the open‐source software R (version 4.2.1). The packages used for this study included “nordpred,” “epitools,” and “INLA.”

## 3 RESULTS

### 3.1 Current hematological tumor burden

**FIGURE 1:**
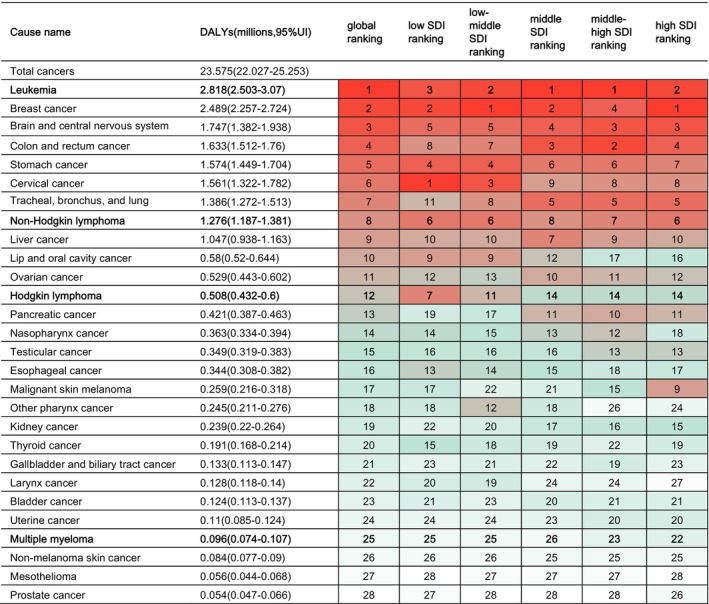
2019 Ranking of total disability‐adjusted life years (DALYs) attributed to cancer in adolescents and young adults, globally and by socio‐demographic index (SDI), for both sexescombined. The bold rows are focuesd hematological malignancies in this study and the transition of colors from red to green to white signifies a ranking from high to low.

This study investigated the burden of cancer, specifically hematological cancers, in AYAs. Figure 1 illustrates the ranking of the total disability‐adjusted life years (DALYs) attributed to cancer in 2019, both globally and according to the SDI. Cancer was responsible for 23.575 million DALYs (95% UI: 22.027–25.253) in 2019. The three leading causes in terms of absolute DALY burden were leukemia, breast cancer, and brain and central nervous system cancer. Among hematological tumors, leukemia accounted for 2.818 million DALYs (95% UI: 2.503–3.07), non‐Hodgkin lymphoma (NHL) for 1.276 million DALYs (95% UI: 1.187–1.381), Hodgkin lymphoma (HL) for 0.508 million DALYs (95% UI: 0.432–0.600), and multiple myeloma (MM) for 0.096 million DALYs (95% UI: 0.074–0.107). These rankings corresponded to the first, 8th, 12th, and 25th positions among all cancers, respectively.

### 3.2 Trends in incidence and deaths over time

Tables 1 and 2 present the incidence, mortality, and trend analysis of the four types of hematological malignancies and subgroups of leukemia from 1990 to 2019 in AYAs. Focusing on leukemia, the incidence in AYAs was 80,834.3 (95% UI: 70,458.9–89,561.5) in 1990 and 101,206.7 (95% UI: 89,857.8–110,493.1) in 2019. The deaths in 1990 and 2019 were 49,563.9 (95% UI: 42,897.8–54,974.5) and 45,105.7 (95% UI: 40,025.5–49,112.9), respectively. The ASIR of leukemia decreased from 3.69/100,000 (95% UI: 3.66–3.71) in 1990 to 3.40/100,000 (95% UI: 3.37–3.42) in 2019, with an EAPC of −0.52 (95% CI: −0.62 to −0.41). The ASDR of leukemia decreased from 2.26/100,000 (95% UI: 2.24–2.28) in 1990 to 1.52/100,000 (95% UI: −1.50–1.03) in 2019, with an EAPC of −1.62 (95% CI: −1.73 to –1.50). Compared with 1990, the ASIR of NHL has increased from 1.45/100,000 (95% UI: 1.44–1.47) to 1.75/100,000 (95% UI:1.74–1.77), with an EAPC of 0.45 (95% UI: 0.36–0.54). On the other hand, the ASDR of NHL has declined. In HL patients, ASIR and ASDR show significant decreases, with EAPC being −0.39 (−0.45 to –0.32) and −1.61 (−1.70 to –1.51), respectively, indicating a more pronounced decline in ASDR. In MM, the incidence was relatively low in the AYA group. As shown in Tables 1 and 2, both the incidence and deaths have increased significantly. Furthermore, ASIR and ASDR showed upward trends, with EAPCs being 0.76 (0.55–0.98) and 0.43 (0.22–0.65), respectively.

**TABLE 1:** The global incident cases, and change trends of hematologic malignancies among AYA from 1990 to 2019.

Cause name	Incidence_1990	ASIR (per 100,000)	Incidence_2019	ASIR (per 100,000)	EAPC_incidence
Hodgkin lymphoma	26,720.7 (21,312.2–28,985.9)	1.23 (1.21–1.24)	33,387.8 (29,927.6–40,538.4)	1.12 (1.11–1.13)	−0.39 (−0.45 to −0.32)
Non‐Hodgkin lymphoma	31,638.8 (28,248.6–35,879.4)	1.45 (1.44–1.47)	52,426.9 (47,048.1–58,728.8)	1.75 (1.74–1.77)	0.45 (0.36–0.54)
Multiple myeloma	1460.5 (1292.5–1918.9)	0.07 (0.07–0.07)	2930.8 (2264.1–3340.2)	0.1 (0.09–0.1)	0.76 (0.55–0.98)
Leukemia	80,834.3 (70,458.9–89,561.5)	3.69 (3.66–3.71)	101,206.7 (89,857.8–110,493.1)	3.4 (3.37–3.42)	−0.52 (−0.62 to −0.41)
Acute lymphoid leukemia	17,615.5 (15,168.5–20,849.7)	0.79 (0.78–0.8)	38,746.5 (32,175.6–43,245.4)	1.31 (1.29–1.32)	1.73 (1.66–1.8)
Acute myeloid leukemia	14,131 (12,506.5–16,221.1)	0.64 (0.63–0.65)	20,182.7 (18,231.5–22,813.6)	0.68 (0.67–0.69)	0.13 (0.1–0.17)
Chronic lymphoid leukemia	1411.2 (1123.4–1684.8)	0.07 (0.06–0.07)	4262.9 (3650.3–4880.5)	0.14 (0.14–0.15)	2.8 (2.58–3.01)
Chronic myeloid leukemia	7800.9 (6665.3–9078.3)	0.36 (0.35–0.37)	9203.2 (8343.8–10,239)	0.3 (0.3–0.31)	−1.19 (−1.39 to −0.99)
Other leukemia	39,875.7 (29,970–46,719.8)	1.83 (1.81–1.84)	28,811.3 (23,837.1–32,787.6)	0.96 (0.95–0.97)	−2.57 (−2.81 to −2.32)

Abbreviations: ASIR, age‐standardized incidence rate; AYA, adolescents and young adults; EAPC, estimated annual percentage change.

**TABLE 2:** The global deaths cases, and change trends of hematologic malignancies among AYA from 1990 to 2019.

Cause name	Deaths_1990	ASDR (per 100,000)	Deaths_2019	ASDR (per 100,000)	EAPC_deaths
Hodgkin lymphoma	8918.2 (6970.4–9860.9)	0.41 (0.4–0.42)	8093.3 (6854.6–9520.6)	0.27 (0.26–0.28)	−1.61 (−1.7 to −1.51)
Non‐Hodgkin lymphoma	15,196.5 (14,006.3–16,428.7)	0.7 (0.69–0.71)	20,797.4 (19,322–22,567.3)	0.69 (0.68–0.7)	−0.25 (−0.32 to −0.17)
Multiple myeloma	952.2 (830.8–1275.1)	0.05 (0.05–0.05)	1680.3 (1307.7–1882.5)	0.06 (0.06–0.06)	0.43 (0.22–0.65)
Leukemia	49,563.9 (42,897.8–54,974.5)	2.26 (2.24–2.28)	45,105.7 (40,025.5–49,112.9)	1.52 (1.5–1.53)	−1.62 (−1.73 to −1.5)
Acute lymphoid leukemia	9970.1 (8517.6–12,133.5)	0.45 (0.44–0.46)	11,696.5 (9635.9–12,916)	0.4 (0.39–0.41)	−0.3 (−0.36 to −0.24)
Acute myeloid leukemia	8889.9 (7797.3–10,176.2)	0.41 (0.4–0.42)	12,166.2 (11,021.2–13,990)	0.41 (0.4–0.42)	−0.06 (−0.11 to −0.01)
Chronic lymphoid leukemia	697.2 (534.7–858.9)	0.04 (0.04–0.04)	1009.7 (871.5–1143)	0.04 (0.04–0.04)	−0.01 (−0.15–0.14)
Chronic myeloid leukemia	5231 (4350.2–6219)	0.24 (0.24–0.25)	4955.6 (4389.3–5623.4)	0.16 (0.16–0.17)	−1.75 (−1.88 to −1.62)
Other leukemia	24,775.7 (18,427.9–29,141.6)	1.13 (1.12–1.15)	15,277.6 (12,687.3–17,359.2)	0.51 (0.5–0.52)	−3.16 (−3.43 to −2.89)

Abbreviations: ASDR, age‐standardized death rate; AYA, adolescents and young adults; EAPC, estimated annual percentage change.

### 3.3 Disease burden of hematological malignancies varies across different regions, different age groups, and different sexes

The disease burden associated with hematological malignancies exhibits notable disparities across different regions and between sexes.^5^ In order to assess this disparity, a comparative analysis of the proportional death rates and proportional incidence rates of hematological malignancies was conducted among various regions and age groups (Figure S1). Leukemia emerged as having the leading incidence and mortality rates among AYAs in all SDI regions and different age groups, accounting for over 50% of cases. Among the 21 GBD regions in the composition of hematological malignancies, Oceania had the highest incidence of leukemia, with a proportional incidence rate of 74.58% and a proportional death rate of 74.49%. In High‐income Australia, HL had the highest proportional incidence rate at 40.35%, while its proportional mortality rate was 10.12%, lower than the rate in Eastern Sub‐Saharan Africa, at 25.30% (Tables S1 and S2).

The age subgroup analysis revealed that MM, a hematological malignancy predominantly affecting older adults, had an incidence rate of 0 in the 15–19 age group. HL showed a higher proportional incidence rate in the 25–29 group compared with the other age groups. In the 15–19 age group, the proportional death rate due to leukemia was the highest, at 69.22%, while in the other age groups, it was 61.08% (20–24 years), 58.77% (25–29 years), 57.28% (30–34 years), and 54.66% (35–39 years) (Tables S3 and S4).

**FIGURE 2:**
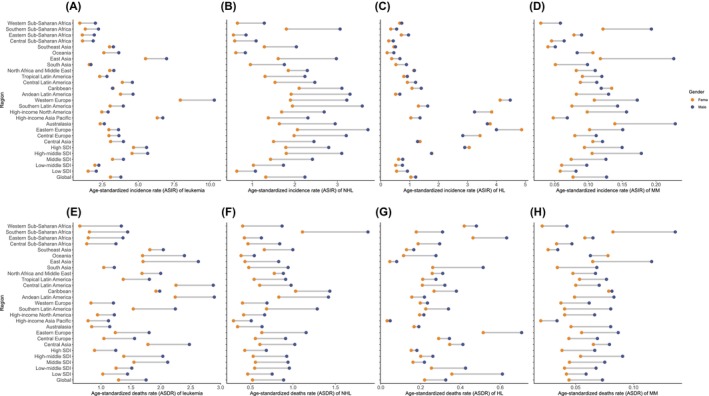
Age‐standardized incidence rate (ASIR) of four hematological tumors by different region and gender in 2019. (A) leukemia; (B) Non‐Hodgkin’s lymphoma; (C) Hodgkin’s lymphoma; (D) multiple myeloma. Age‐standardized death rate (ASDR) of four hematological tumors by different region and gender in 2019. (E) leukemia; (F) Non‐Hodgkin’s lymphoma; (G) Hodgkin’s lymphoma; (H) multiple myeloma.

Furthermore, the disease burden demonstrated significant variations across regions and nations based on the SDI levels and 21 GBD regions (Figure 2). Compared with countries with low SDI, those in the higher SDI quintiles exhibited notably higher ASIR for hematological malignancies among the AYA population. Conversely, the ASDR displayed a marked decrease with increasing SDI. Notably, across all hematological malignancies in different SDI regions, the ASIR is consistently higher for males than females among the AYA population (Figure 2). In the 21 GBD regions, Western Europe had the highest ASIR for leukemia, with rates of 10.29 per 100,000 for females and 7.93 per 100,000 for males. On the other hand, it had a relatively lower ASDR of 1.21 per 100,000 for males and 0.83 per 100,000 for females, ranking 16th and 19th, respectively. Furthermore, although there was no highest ASIR (ranking 15th for males and 14th for females among the 21 GBD regions), Southern Sub‐Saharan Africa had the highest ASDR, reaching 1.88 per 100,000 for males and 1.11 per 100,000 for females.

### 3.4 Burden prediction of hematological malignancies

**FIGURE 3:**
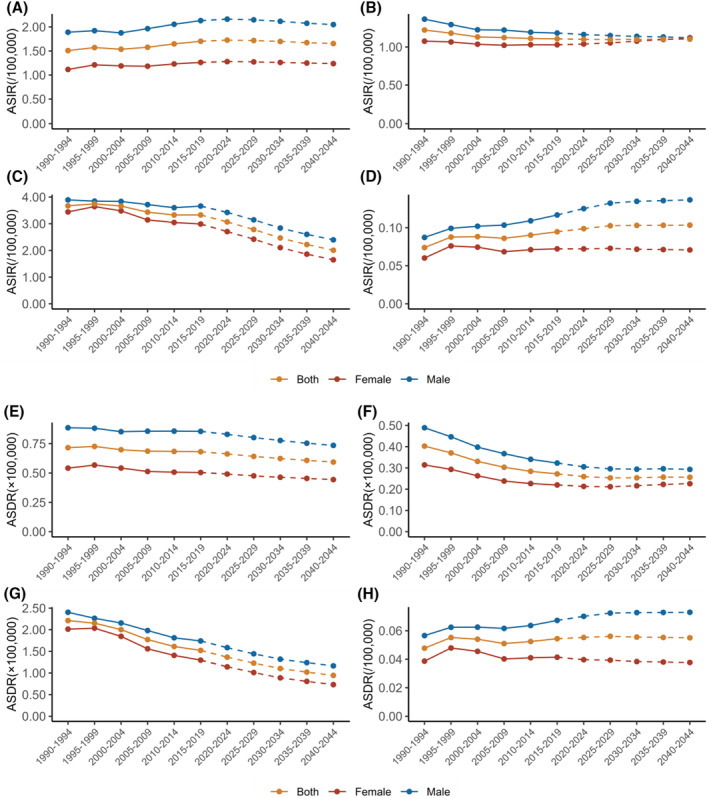
Trends in age‐standardized incidence rate (ASIR) of hematological malignancies, from 1990 to 2044. (A) Non‐Hodgkin's lymphoma; (B) Hodgkin's lymphoma; (C) leukemia; (D) multiple myeloma. Trends in age‐standardized death rate (ASDR) of hematological malignancies, from 1990 to 2044. (E) Non‐Hodgkin's lymphoma; (F) Hodgkin's lymphoma; (G) leukemia; (H) multiple myeloma.

The historical and projected trends of ASIR and ASDR for hematological malignancies in AYAs were analyzed (Figure 3). The incidence of leukemia has been declining annually, with a projected ASIR of 1.65/100,000 for females and 2.40/100,000 for males by 2044. Simultaneously, the mortality rate of leukemia has been declining every year. By 2039–2044, the ASDR is expected to decrease to 0.94/100,000. On the other hand, the incidence of NHL has been gradually increasing in recent years, with an ASIR of 1.73/100,000 from 2020 to 2024, but the ASDR of NHL decreased year by year, with the highest value in the period of 1990–1994 (0.73/100,000). The ASIR of HL among males has been declining steadily over the years and is projected to reach 1.12/100,000 by 2040–2044. On the other hand, among females, the ASIR declined from 1990 to 2009 but has shown an upward trend in recent years. In addition, in AYA patients, the less common MM shows a divergent trend in ASIR between males and females. The incidence among males has been increasing, while among females, it has decreased. The ASDR also exhibited a similar trend, indicating higher mortality rates for males and lower mortality rates for females over time (Tables S5 and S6).

The incidence and mortality trends of hematological malignancies in different age groups were analyzed. The AYAs were divided into five age groups: 15–19, 20–24, 25–29, 30–34, and 35–39 years. The incidence rate and mortality of four types of hematological malignancies in each age group are shown in Figure S2. Among the four hematological malignancies, except for HL, the highest incidence occurred in the 35–39 age group, significantly higher than in the other age groups. During 2015–2019, NHL, leukemia, and MM had incidence rates (per 100,000) of 1.024, 1.811 and 0.149, respectively, for the 35–39 age group. HL in the 15–19 age group was significantly lower than in the other age groups, while the age group of 20–39 years showed similar incidence rates. The age group with the highest incidence of HL was 25–59 years old, with a rate of 0.413 per 100,000 population during 2015–2019. MM is considered a geriatric disease, with a median age of onset around 70 years.^19^ The analysis results show that the incidence of MM in the 15–19 age group was nearly 0. However, there was a significant increase in incidence in the age group of 34–39 years. As for NHL, in the 15–19 age group, the incidence rate was almost comparable to the 30–34 age group and higher than in the 20–29 age group, but the mortality rate was the lowest. In the case of HL, adolescents aged 15–19 had the lowest incidence and mortality rates. On the other hand, for leukemia, the incidence and mortality rates in adolescents aged 15–19 years were similar to those in the 20–34 age group (Tables S7 and S8).

**FIGURE 4:**
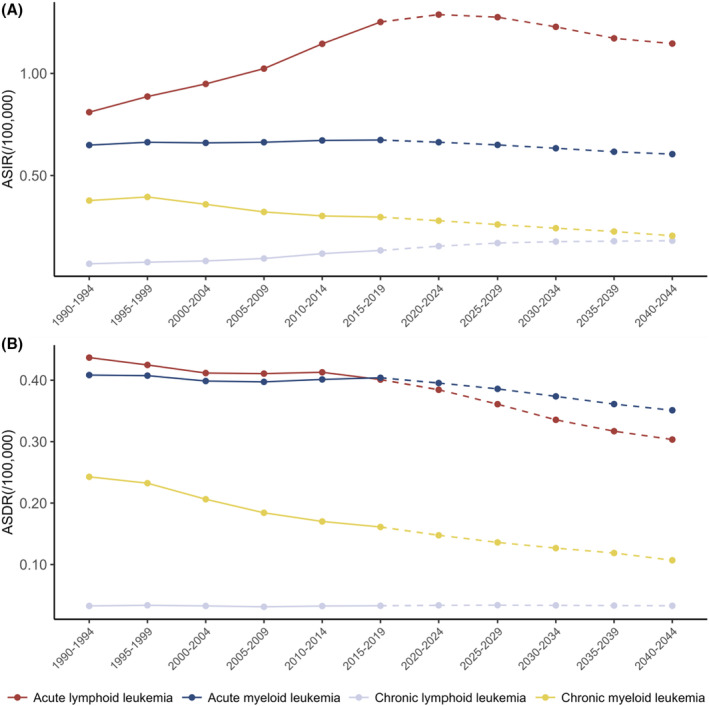
Trends in (A) age‐standardized incidence rate (ASIR) and (B) age‐standardized death rate (ASDR): Subgroup analysis of leukemia from 1990 to 2044.

The ASIR and ASDR of different leukemia types in the AYA population were analyzed and presented in Figure 4. Chronic lymphocytic leukemia has a very low incidence and mortality rate in the AYA population. Among the four leukemia subtypes, the highest incidence in AYAs was observed for acute lymphocytic leukemia (ALL), peaking from 2020 to 2024 at 1.29/100,000. Still, in the predictive model, the ASDR of ALL was expected to gradually become lower than in patients with acute myeloid leukemia (AML). During 2040–2044, the ASDR for the 15–39 age group is projected to be 0.303 for ALL and 0.351 for AML. In AYAs, the ASIR of chronic myeloid leukemia (CML) has gradually decreased over the years, but the decline in ASDR was even more pronounced. It is projected to decrease from 0.24/100,000 in 1990–1994 to 0.11/100,000 by 2040–2044 (Tables S9 and S10).

## 4 DISCUSSION

In recent years, the unique challenges associated with managing cancer in AYA have become increasingly acknowledged.^4^, ^20^, ^21^, ^22^, ^23^ Previous studies on the burden of cancer in the AYA population have shown that the majority of the increase was attributed to the overdiagnosis of thyroid cancer. However, tumors of the lymphatic and hematopoietic systems also pose a significant threat to the AYA population.^20^, ^24^ This study aimed to analyze the changes in the disease burden of hematological malignancies among the global AYA over the past three decades based on the GBD database. Leukemia has the highest burden of all types of hematologic malignancies in this age group.^5^, ^25^ The present study revealed that leukemia ranks first as the leading tumor burden among the global AYA population. Meanwhile, similar to previous reports, the ASDR of leukemia was decreasing year by year during the study period, largely attributed to advancements in diagnosis and treatment.^26^, ^27^ In recent years, in addition to traditional morphological methods such as bone marrow routine staining, the utilization of techniques like flow cytometry and molecular genetics has significantly improved the accuracy of diagnosis.^28^, ^29^ On the other hand, leukemia patients have experienced a substantial reduction in mortality rates, owing to the continuous development of novel medications, supportive treatment, and the implementation of advanced treatments like immunotherapy and targeted therapy. For example, Huo et al. reported an overall 3‐year survival rate of 85.6% in patients with AML aged 15–39 years who underwent hematopoietic stem cell transplantation.^30^ Furthermore, in recent years, there has been a significant decline in the mortality rate of ALL in the AYA population. It is even projected that in the future, the mortality rate of ALL might become lower than that of AML. It could be due to changes in treatment approaches for this age group, particularly implementing pediatric regimens. A specialized study on the disease burden of ALL in the AYA population also reached similar conclusions.^31^ A large prospective clinical trial showed that by using pediatric treatment protocols in patients with ALL, significant advancements have been observed in the AYA population. The overall survival (OS) at 3 years was 73%, superior to earlier Cancer and Leukemia Group B (CALGB) studies, where the OS for this age group was 55% at 3 years.^32^, ^33^ Furthermore, in the predictive model, the future incidence rate of leukemia is also decreasing year by year. A potential explanation for this trend could be decreased occupational exposure and increased health awareness.^16^

Similar to previous studies, the incidence rate of NHL continues to increase.^34^ The application of immunotherapeutic agents such as rituximab has significantly improved the survival of the patients. The application of positron emission tomography‐computed tomography in NHL has provided a powerful help in the early detection of the disease and recurrences and precise treatment.^35^ MM, a disease commonly occurring in older adults,^36^ has shown an increasing incidence rate among younger individuals. While part of this trend can be attributed to improved detection rates, numerous underlying factors still require further exploration. In a study by Ravi et al.,^37^ the OS for patients under 50 with MM remained relatively low compared with HL and diffuse large B‐cell lymphoma (DLBCL). The 3‐year OS rate for MM was 75.6%, resulting in a 20‐fold excess mortality risk in the under 50 age group, while the excess mortality risks for HL and DLBCL were 0.9 and 3.1, respectively. Therefore, the treatment of MM in AYA still requires more attention.^38^ According to the classification by the International Agency for Research on Cancer in 2016, there is strong evidence indicating that excess body weight is causally linked to 12 types of cancer, including multiple myeloma.^39^ Preventing or treating obesity during childhood and early adulthood could potentially reduce future rises.

Regional analysis based on the SDI indicates that in low‐ and middle‐SDI regions, there is still an urgent need for improvement in the diagnosis and treatment of hematological malignancies. Leukemia ranks fifth in terms of economic costs in a global cancer cost estimation, but in certain low‐to‐middle SDI countries, it represents the highest macroeconomic burden.^38^ Thus, allocating sufficient medical resources and establishing robust healthcare systems in low‐ and middle‐income countries is imperative. This study also showed that in regions with different levels of economic development, almost all hematologic malignancies had higher incidence rates among males than females. It may be attributed to unfavorable lifestyle habits and occupational exposures.^16^, ^40^, ^41^ Furthermore, by comparing the ASIR and ASDR across 21 different regions, the ASIR of leukemia in Western Europe was higher than in the other regions. It prompted a further exploration of the specific causes to seek disease prevention strategies. NHL had the highest ASDR in Southern Sub‐Saharan Africa, necessitating corresponding medical assistance and policy support to enhance the local diagnosis and treatment capabilities for NHL.

Considering the variations in disease burden, distinct disease prevention and control strategies be adopted while future health policies are being formulated. First, despite leukemia remaining the leading hematological cancer burden among AYA, the increasing incidence rate of NHL necessitates our attention. Second, in low and low‐middle‐income countries, efforts should continue to improve the diagnosis and treatment of hematological malignancies while requiring global‐level healthcare assistance. Furthermore, with the improvement in treatment outcomes and the advent of the chemotherapy‐free era, societies should also focus on the long‐term management of the patients,^42^ guiding them towards reemployment and enabling them to lead normal work and life.^24^, ^43^, ^44^ Finally, primary prevention plays a crucial role in alleviating the burden of cancer, reducing both suffering and mortality rates, and lowering healthcare costs associated with cancer treatment.^22^ Therefore, it should be recognized as an indispensable component of comprehensive cancer control efforts today and in the future.

This study had some limitations, including the potential diagnostic accuracy and ascertainment biases that are inherent to all cancer registries. These limitations could have impacted the estimation of cancer mortality and DALYs in the GBD study, stemming from the misclassification and miscoding of deaths.^2^, ^3^ A comprehensive analysis of the global trends in the disease burden of hematological malignancies was conducted, but the primary causes leading to these changes could not be analyzed. Further exploration and analysis are needed. The models were based on past incidence data and the future age structure of the population without considering unknown environmental changes and variations in disease occurrence and mortality caused by new technological advancements. Additionally, the data used for the prediction comes from the GBD 2019 database and does not take the impact of COVID‐19 into account. A recent study indicates that in 2021, COVID‐19 was the leading cause of DALYs globally.^45^ However, precisely because of this, by comparing the predicted ASIR and ASDR, we can assess the impact of COVID‐19 infection on patient prognosis.

In conclusion, the ASIR of leukemia shows a decreasing trend, while the ASIR of NHL shows an increasing trend in AYA. The overall prognosis for patients with hematologic malignancies is gradually improving. Consequently, for the AYA population, it is essential to focus on long‐term management, psychological support, and employment promotion.

## AUTHOR CONTRIBUTIONS

**Linlin Huang:** Investigation (equal); methodology (equal); writing – original draft (lead). **Jingsong He:** Funding acquisition (lead); investigation (equal); methodology (equal).

## CONFLICT OF INTEREST STATEMENT

The authors declare no competing financial interests.

## Supporting information


Data S1.

## Data Availability

The data that support the findings of this study are openly available in https://vizhub.healthdata.org/gbd‐results/, https://population.un.org/wpp/Download/Standard/Population/, and https://seer.cancer.gov/stdpopulations/world.who.html.
