# Supplementary material for: Trend analysis of hematological tumors in adolescents and young adults from 1990 to 2019 and predictive trends from 2020 to 2044: A Global Burden of Disease study
Source: Cancer Med. 2024 Oct 3;13(18):e70224. doi: 10.1002/cam4.70224 (PMC11447274; doi:10.1002/cam4.70224)
Supplement: Supplementary file 1 — Data S1. [file CAM4-13-e70224-s001.docx]

**Table S 1** Proportional burden of incidence for hematological malignancies in adolescents and young adults across various regions in 2019

| location_name | cause_name | n | percentage |
| --- | --- | --- | --- |
| Global | Hodgkin lymphoma | 31446.73 | 0.180265 |
| Low SDI | Hodgkin lymphoma | 2461.887 | 0.213029 |
| Low-middle SDI | Hodgkin lymphoma | 3905.501 | 0.150047 |
| Middle SDI | Hodgkin lymphoma | 5451.581 | 0.10097 |
| High-middle SDI | Hodgkin lymphoma | 9094.999 | 0.193139 |
| High SDI | Hodgkin lymphoma | 10514.53 | 0.294717 |
| Central Asia | Hodgkin lymphoma | 428.5715 | 0.179323 |
| Central Europe | Hodgkin lymphoma | 1232.937 | 0.339681 |
| Eastern Europe | Hodgkin lymphoma | 2996.604 | 0.407234 |
| Australasia | Hodgkin lymphoma | 327.4108 | 0.403521 |
| High-income Asia Pacific | Hodgkin lymphoma | 769.384 | 0.13021 |
| High-income North America | Hodgkin lymphoma | 5007.162 | 0.432579 |
| Southern Latin America | Hodgkin lymphoma | 290.4851 | 0.163121 |
| Western Europe | Hodgkin lymphoma | 6115.701 | 0.271274 |
| Andean Latin America | Hodgkin lymphoma | 122.8534 | 0.074306 |
| Caribbean | Hodgkin lymphoma | 219.9576 | 0.190091 |
| Central Latin America | Hodgkin lymphoma | 884.2435 | 0.134186 |
| Tropical Latin America | Hodgkin lymphoma | 737.1204 | 0.161407 |
| North Africa and Middle East | Hodgkin lymphoma | 2262.276 | 0.164949 |
| South Asia | Hodgkin lymphoma | 4517.695 | 0.191069 |
| East Asia | Hodgkin lymphoma | 2310.053 | 0.053067 |
| Oceania | Hodgkin lymphoma | 14.04259 | 0.07744 |
| Southeast Asia | Hodgkin lymphoma | 1121.166 | 0.083961 |
| Central Sub-Saharan Africa | Hodgkin lymphoma | 127.4105 | 0.130608 |
| Eastern Sub-Saharan Africa | Hodgkin lymphoma | 1018.108 | 0.264088 |
| Southern Sub-Saharan Africa | Hodgkin lymphoma | 135.5028 | 0.090146 |
| Western Sub-Saharan Africa | Hodgkin lymphoma | 808.0493 | 0.217015 |
| Global | Non-Hodgkin lymphoma | 45288.44 | 0.259611 |
| Low SDI | Non-Hodgkin lymphoma | 2574.502 | 0.222774 |
| Low-middle SDI | Non-Hodgkin lymphoma | 7354.144 | 0.282542 |
| Middle SDI | Non-Hodgkin lymphoma | 15000.81 | 0.277835 |
| High-middle SDI | Non-Hodgkin lymphoma | 12125.88 | 0.257503 |
| High SDI | Non-Hodgkin lymphoma | 8205.043 | 0.229983 |
| Central Asia | Non-Hodgkin lymphoma | 617.6468 | 0.258436 |
| Central Europe | Non-Hodgkin lymphoma | 1068.292 | 0.29432 |
| Eastern Europe | Non-Hodgkin lymphoma | 2025.667 | 0.275285 |
| Australasia | Non-Hodgkin lymphoma | 229.577 | 0.282945 |
| High-income Asia Pacific | Non-Hodgkin lymphoma | 1317.866 | 0.223035 |
| High-income North America | Non-Hodgkin lymphoma | 2952.81 | 0.255099 |
| Southern Latin America | Non-Hodgkin lymphoma | 652.6759 | 0.366508 |
| Western Europe | Non-Hodgkin lymphoma | 3933.215 | 0.174465 |
| Andean Latin America | Non-Hodgkin lymphoma | 513.2835 | 0.310452 |
| Caribbean | Non-Hodgkin lymphoma | 399.7452 | 0.345467 |
| Central Latin America | Non-Hodgkin lymphoma | 1726.965 | 0.26207 |
| Tropical Latin America | Non-Hodgkin lymphoma | 1464.38 | 0.320655 |
| North Africa and Middle East | Non-Hodgkin lymphoma | 3994.35 | 0.291239 |
| South Asia | Non-Hodgkin lymphoma | 7493.119 | 0.31691 |
| East Asia | Non-Hodgkin lymphoma | 10544.75 | 0.242238 |
| Oceania | Non-Hodgkin lymphoma | 28.52023 | 0.157279 |
| Southeast Asia | Non-Hodgkin lymphoma | 3564.893 | 0.266964 |
| Central Sub-Saharan Africa | Non-Hodgkin lymphoma | 253.5037 | 0.259866 |
| Eastern Sub-Saharan Africa | Non-Hodgkin lymphoma | 786.1398 | 0.203918 |
| Southern Sub-Saharan Africa | Non-Hodgkin lymphoma | 698.6036 | 0.46476 |
| Western Sub-Saharan Africa | Non-Hodgkin lymphoma | 1022.436 | 0.274593 |
| Global | Multiple myeloma | 2429.364 | 0.013926 |
| Low SDI | Multiple myeloma | 178.9783 | 0.015487 |
| Low-middle SDI | Multiple myeloma | 402.624 | 0.015469 |
| Middle SDI | Multiple myeloma | 745.294 | 0.013804 |
| High-middle SDI | Multiple myeloma | 660.217 | 0.01402 |
| High SDI | Multiple myeloma | 440.8672 | 0.012357 |
| Central Asia | Multiple myeloma | 30.7595 | 0.01287 |
| Central Europe | Multiple myeloma | 40.59971 | 0.011185 |
| Eastern Europe | Multiple myeloma | 84.87619 | 0.011535 |
| Australasia | Multiple myeloma | 18.79605 | 0.023165 |
| High-income Asia Pacific | Multiple myeloma | 45.43092 | 0.007689 |
| High-income North America | Multiple myeloma | 171.9234 | 0.014853 |
| Southern Latin America | Multiple myeloma | 21.68554 | 0.012177 |
| Western Europe | Multiple myeloma | 225.449 | 0.01 |
| Andean Latin America | Multiple myeloma | 19.6594 | 0.011891 |
| Caribbean | Multiple myeloma | 18.54345 | 0.016026 |
| Central Latin America | Multiple myeloma | 72.00541 | 0.010927 |
| Tropical Latin America | Multiple myeloma | 79.46801 | 0.017401 |
| North Africa and Middle East | Multiple myeloma | 167.8563 | 0.012239 |
| South Asia | Multiple myeloma | 413.1659 | 0.017474 |
| East Asia | Multiple myeloma | 741.7574 | 0.01704 |
| Oceania | Multiple myeloma | 3.454835 | 0.019052 |
| Southeast Asia | Multiple myeloma | 107.7493 | 0.008069 |
| Central Sub-Saharan Africa | Multiple myeloma | 14.55909 | 0.014924 |
| Eastern Sub-Saharan Africa | Multiple myeloma | 72.88846 | 0.018907 |
| Southern Sub-Saharan Africa | Multiple myeloma | 38.84652 | 0.025843 |
| Western Sub-Saharan Africa | Multiple myeloma | 39.88996 | 0.010713 |
| Global | Acute lymphoid leukemia | 30448.71 | 0.174544 |
| Low SDI | Acute lymphoid leukemia | 1304.547 | 0.112884 |
| Low-middle SDI | Acute lymphoid leukemia | 3003.412 | 0.11539 |
| Middle SDI | Acute lymphoid leukemia | 7660.186 | 0.141877 |
| High-middle SDI | Acute lymphoid leukemia | 9471.031 | 0.201125 |
| High SDI | Acute lymphoid leukemia | 8994.012 | 0.252098 |
| Central Asia | Acute lymphoid leukemia | 285.8209 | 0.119593 |
| Central Europe | Acute lymphoid leukemia | 463.8548 | 0.127795 |
| Eastern Europe | Acute lymphoid leukemia | 904.318 | 0.122895 |
| Australasia | Acute lymphoid leukemia | 94.85352 | 0.116903 |
| High-income Asia Pacific | Acute lymphoid leukemia | 2498.137 | 0.422783 |
| High-income North America | Acute lymphoid leukemia | 1271.136 | 0.109816 |
| Southern Latin America | Acute lymphoid leukemia | 268.4671 | 0.150757 |
| Western Europe | Acute lymphoid leukemia | 8194.845 | 0.363498 |
| Andean Latin America | Acute lymphoid leukemia | 303.5668 | 0.183608 |
| Caribbean | Acute lymphoid leukemia | 111.7319 | 0.096561 |
| Central Latin America | Acute lymphoid leukemia | 1698.543 | 0.257757 |
| Tropical Latin America | Acute lymphoid leukemia | 643.8465 | 0.140983 |
| North Africa and Middle East | Acute lymphoid leukemia | 1576.124 | 0.114919 |
| South Asia | Acute lymphoid leukemia | 2602.15 | 0.110054 |
| East Asia | Acute lymphoid leukemia | 7190.461 | 0.165182 |
| Oceania | Acute lymphoid leukemia | 14.28476 | 0.078776 |
| Southeast Asia | Acute lymphoid leukemia | 1390.307 | 0.104116 |
| Central Sub-Saharan Africa | Acute lymphoid leukemia | 82.73932 | 0.084816 |
| Eastern Sub-Saharan Africa | Acute lymphoid leukemia | 468.3588 | 0.121488 |
| Southern Sub-Saharan Africa | Acute lymphoid leukemia | 84.20293 | 0.056018 |
| Western Sub-Saharan Africa | Acute lymphoid leukemia | 300.9582 | 0.080827 |
| Global | Acute myeloid leukemia | 18745.64 | 0.107457 |
| Low SDI | Acute myeloid leukemia | 1527.42 | 0.132169 |
| Low-middle SDI | Acute myeloid leukemia | 3568.191 | 0.137088 |
| Middle SDI | Acute myeloid leukemia | 6149.92 | 0.113905 |
| High-middle SDI | Acute myeloid leukemia | 4263.205 | 0.090533 |
| High SDI | Acute myeloid leukemia | 3222.58 | 0.090327 |
| Central Asia | Acute myeloid leukemia | 392.616 | 0.164279 |
| Central Europe | Acute myeloid leukemia | 415.0338 | 0.114344 |
| Eastern Europe | Acute myeloid leukemia | 587.8509 | 0.079888 |
| Australasia | Acute myeloid leukemia | 81.4449 | 0.100378 |
| High-income Asia Pacific | Acute myeloid leukemia | 535.5703 | 0.090639 |
| High-income North America | Acute myeloid leukemia | 1106.775 | 0.095617 |
| Southern Latin America | Acute myeloid leukemia | 251.9705 | 0.141493 |
| Western Europe | Acute myeloid leukemia | 1507.034 | 0.066847 |
| Andean Latin America | Acute myeloid leukemia | 219.0763 | 0.132505 |
| Caribbean | Acute myeloid leukemia | 147.0379 | 0.127073 |
| Central Latin America | Acute myeloid leukemia | 1009.943 | 0.153261 |
| Tropical Latin America | Acute myeloid leukemia | 930.924 | 0.203845 |
| North Africa and Middle East | Acute myeloid leukemia | 2300.497 | 0.167735 |
| South Asia | Acute myeloid leukemia | 3511.96 | 0.148533 |
| East Asia | Acute myeloid leukemia | 2626.453 | 0.060336 |
| Oceania | Acute myeloid leukemia | 53.23167 | 0.293554 |
| Southeast Asia | Acute myeloid leukemia | 1909.486 | 0.142996 |
| Central Sub-Saharan Africa | Acute myeloid leukemia | 94.11043 | 0.096472 |
| Eastern Sub-Saharan Africa | Acute myeloid leukemia | 378.4637 | 0.09817 |
| Southern Sub-Saharan Africa | Acute myeloid leukemia | 127.3967 | 0.084753 |
| Western Sub-Saharan Africa | Acute myeloid leukemia | 558.7626 | 0.150065 |
| Global | Chronic lymphoid leukemia | 2951.31 | 0.016918 |
| Low SDI | Chronic lymphoid leukemia | 69.67546 | 0.006029 |
| Low-middle SDI | Chronic lymphoid leukemia | 247.9626 | 0.009527 |
| Middle SDI | Chronic lymphoid leukemia | 1242.41 | 0.023011 |
| High-middle SDI | Chronic lymphoid leukemia | 1012.291 | 0.021497 |
| High SDI | Chronic lymphoid leukemia | 378.1762 | 0.0106 |
| Central Asia | Chronic lymphoid leukemia | 35.50887 | 0.014858 |
| Central Europe | Chronic lymphoid leukemia | 63.49816 | 0.017494 |
| Eastern Europe | Chronic lymphoid leukemia | 107.6949 | 0.014636 |
| Australasia | Chronic lymphoid leukemia | 8.553834 | 0.010542 |
| High-income Asia Pacific | Chronic lymphoid leukemia | 50.81421 | 0.0086 |
| High-income North America | Chronic lymphoid leukemia | 100.0389 | 0.008643 |
| Southern Latin America | Chronic lymphoid leukemia | 9.809887 | 0.005509 |
| Western Europe | Chronic lymphoid leukemia | 220.667 | 0.009788 |
| Andean Latin America | Chronic lymphoid leukemia | 15.91094 | 0.009623 |
| Caribbean | Chronic lymphoid leukemia | 8.4593 | 0.007311 |
| Central Latin America | Chronic lymphoid leukemia | 45.16452 | 0.006854 |
| Tropical Latin America | Chronic lymphoid leukemia | 22.42014 | 0.004909 |
| North Africa and Middle East | Chronic lymphoid leukemia | 175.3372 | 0.012784 |
| South Asia | Chronic lymphoid leukemia | 147.566 | 0.006241 |
| East Asia | Chronic lymphoid leukemia | 1794.769 | 0.04123 |
| Oceania | Chronic lymphoid leukemia | 0.706755 | 0.003898 |
| Southeast Asia | Chronic lymphoid leukemia | 65.98265 | 0.004941 |
| Central Sub-Saharan Africa | Chronic lymphoid leukemia | 7.303988 | 0.007487 |
| Eastern Sub-Saharan Africa | Chronic lymphoid leukemia | 14.76933 | 0.003831 |
| Southern Sub-Saharan Africa | Chronic lymphoid leukemia | 34.7447 | 0.023115 |
| Western Sub-Saharan Africa | Chronic lymphoid leukemia | 21.59017 | 0.005798 |
| Global | Chronic myeloid leukemia | 8768.879 | 0.050267 |
| Low SDI | Chronic myeloid leukemia | 1339.019 | 0.115867 |
| Low-middle SDI | Chronic myeloid leukemia | 2067.992 | 0.079451 |
| Middle SDI | Chronic myeloid leukemia | 1687.991 | 0.031264 |
| High-middle SDI | Chronic myeloid leukemia | 1576.744 | 0.033483 |
| High SDI | Chronic myeloid leukemia | 2092.129 | 0.058641 |
| Central Asia | Chronic myeloid leukemia | 64.37734 | 0.026937 |
| Central Europe | Chronic myeloid leukemia | 101.5428 | 0.027976 |
| Eastern Europe | Chronic myeloid leukemia | 223.1474 | 0.030325 |
| Australasia | Chronic myeloid leukemia | 29.3824 | 0.036213 |
| High-income Asia Pacific | Chronic myeloid leukemia | 429.0934 | 0.072619 |
| High-income North America | Chronic myeloid leukemia | 368.695 | 0.031852 |
| Southern Latin America | Chronic myeloid leukemia | 40.93734 | 0.022988 |
| Western Europe | Chronic myeloid leukemia | 1684.353 | 0.074713 |
| Andean Latin America | Chronic myeloid leukemia | 41.42489 | 0.025055 |
| Caribbean | Chronic myeloid leukemia | 40.80551 | 0.035265 |
| Central Latin America | Chronic myeloid leukemia | 165.743 | 0.025152 |
| Tropical Latin America | Chronic myeloid leukemia | 140.3067 | 0.030723 |
| North Africa and Middle East | Chronic myeloid leukemia | 699.5985 | 0.05101 |
| South Asia | Chronic myeloid leukemia | 2830.997 | 0.119733 |
| East Asia | Chronic myeloid leukemia | 615.1114 | 0.014131 |
| Oceania | Chronic myeloid leukemia | 9.159236 | 0.05051 |
| Southeast Asia | Chronic myeloid leukemia | 391.6606 | 0.02933 |
| Central Sub-Saharan Africa | Chronic myeloid leukemia | 64.93961 | 0.066569 |
| Eastern Sub-Saharan Africa | Chronic myeloid leukemia | 540.1692 | 0.140115 |
| Southern Sub-Saharan Africa | Chronic myeloid leukemia | 19.26349 | 0.012815 |
| Western Sub-Saharan Africa | Chronic myeloid leukemia | 268.17 | 0.072022 |
| Global | Other leukemia | 34368.48 | 0.197013 |
| Low SDI | Other leukemia | 2100.528 | 0.181761 |
| Low-middle SDI | Other leukemia | 5478.637 | 0.210486 |
| Middle SDI | Other leukemia | 16053.67 | 0.297335 |
| High-middle SDI | Other leukemia | 8885.952 | 0.1887 |
| High SDI | Other leukemia | 1829.364 | 0.051276 |
| Central Asia | Other leukemia | 534.6401 | 0.223704 |
| Central Europe | Other leukemia | 243.9352 | 0.067205 |
| Eastern Europe | Other leukemia | 428.2785 | 0.058202 |
| Australasia | Other leukemia | 21.36547 | 0.026332 |
| High-income Asia Pacific | Other leukemia | 262.5028 | 0.044426 |
| High-income North America | Other leukemia | 596.6025 | 0.051542 |
| Southern Latin America | Other leukemia | 244.7655 | 0.137447 |
| Western Europe | Other leukemia | 663.1226 | 0.029414 |
| Andean Latin America | Other leukemia | 417.5697 | 0.252561 |
| Caribbean | Other leukemia | 210.836 | 0.182208 |
| Central Latin America | Other leukemia | 987.0981 | 0.149794 |
| Tropical Latin America | Other leukemia | 548.3674 | 0.120076 |
| North Africa and Middle East | Other leukemia | 2538.996 | 0.185125 |
| South Asia | Other leukemia | 2127.635 | 0.089985 |
| East Asia | Other leukemia | 17707.18 | 0.406776 |
| Oceania | Other leukemia | 57.93487 | 0.319491 |
| Southeast Asia | Other leukemia | 4802.219 | 0.359623 |
| Central Sub-Saharan Africa | Other leukemia | 330.9496 | 0.339256 |
| Eastern Sub-Saharan Africa | Other leukemia | 576.2825 | 0.149483 |
| Southern Sub-Saharan Africa | Other leukemia | 364.5871 | 0.242549 |
| Western Sub-Saharan Africa | Other leukemia | 703.6075 | 0.188966 |

**Table S 2** Proportional burden of deaths for hematological malignancies in adolescents and young adults across various regions in 2019

| location_name | cause_name | n | percentage |
| --- | --- | --- | --- |
| Global | Hodgkin lymphoma | 8174.244 | 0.106797 |
| Low SDI | Hodgkin lymphoma | 1672.764 | 0.208341 |
| Low-middle SDI | Hodgkin lymphoma | 2329.378 | 0.140892 |
| Middle SDI | Hodgkin lymphoma | 1971.54 | 0.069659 |
| High-middle SDI | Hodgkin lymphoma | 1538.533 | 0.092241 |
| High SDI | Hodgkin lymphoma | 657.3406 | 0.094621 |
| Central Asia | Hodgkin lymphoma | 159.5139 | 0.125085 |
| Central Europe | Hodgkin lymphoma | 169.4233 | 0.146806 |
| Eastern Europe | Hodgkin lymphoma | 559.9894 | 0.217617 |
| Australasia | Hodgkin lymphoma | 17.67213 | 0.101233 |
| High-income Asia Pacific | Hodgkin lymphoma | 27.84602 | 0.026065 |
| High-income North America | Hodgkin lymphoma | 305.8636 | 0.118394 |
| Southern Latin America | Hodgkin lymphoma | 69.65411 | 0.086266 |
| Western Europe | Hodgkin lymphoma | 359.5773 | 0.12378 |
| Andean Latin America | Hodgkin lymphoma | 50.70222 | 0.053541 |
| Caribbean | Hodgkin lymphoma | 58.21395 | 0.10132 |
| Central Latin America | Hodgkin lymphoma | 259.7462 | 0.074663 |
| Tropical Latin America | Hodgkin lymphoma | 249.3652 | 0.103607 |
| North Africa and Middle East | Hodgkin lymphoma | 772.5294 | 0.106552 |
| South Asia | Hodgkin lymphoma | 2801.87 | 0.183872 |
| East Asia | Hodgkin lymphoma | 464.5666 | 0.024312 |
| Oceania | Hodgkin lymphoma | 8.393075 | 0.071043 |
| Southeast Asia | Hodgkin lymphoma | 450.8228 | 0.056505 |
| Central Sub-Saharan Africa | Hodgkin lymphoma | 88.30589 | 0.127725 |
| Eastern Sub-Saharan Africa | Hodgkin lymphoma | 688.2023 | 0.252975 |
| Southern Sub-Saharan Africa | Hodgkin lymphoma | 82.11132 | 0.080676 |
| Western Sub-Saharan Africa | Hodgkin lymphoma | 529.8747 | 0.214704 |
| Global | Non-Hodgkin lymphoma | 19215.95 | 0.251058 |
| Low SDI | Non-Hodgkin lymphoma | 1912.95 | 0.238256 |
| Low-middle SDI | Non-Hodgkin lymphoma | 4456.122 | 0.269527 |
| Middle SDI | Non-Hodgkin lymphoma | 6703.243 | 0.236842 |
| High-middle SDI | Non-Hodgkin lymphoma | 4052.016 | 0.242933 |
| High SDI | Non-Hodgkin lymphoma | 2078.35 | 0.299169 |
| Central Asia | Non-Hodgkin lymphoma | 279.1423 | 0.218893 |
| Central Europe | Non-Hodgkin lymphoma | 338.8017 | 0.293572 |
| Eastern Europe | Non-Hodgkin lymphoma | 706.7131 | 0.274635 |
| Australasia | Non-Hodgkin lymphoma | 50.76614 | 0.29081 |
| High-income Asia Pacific | Non-Hodgkin lymphoma | 313.7125 | 0.293642 |
| High-income North America | Non-Hodgkin lymphoma | 738.9909 | 0.28605 |
| Southern Latin America | Non-Hodgkin lymphoma | 250.3884 | 0.310104 |
| Western Europe | Non-Hodgkin lymphoma | 885.4745 | 0.304814 |
| Andean Latin America | Non-Hodgkin lymphoma | 253.3439 | 0.267531 |
| Caribbean | Non-Hodgkin lymphoma | 187.0172 | 0.325499 |
| Central Latin America | Non-Hodgkin lymphoma | 722.9625 | 0.207813 |
| Tropical Latin America | Non-Hodgkin lymphoma | 659.0393 | 0.273821 |
| North Africa and Middle East | Non-Hodgkin lymphoma | 1822.725 | 0.251401 |
| South Asia | Non-Hodgkin lymphoma | 4416.476 | 0.28983 |
| East Asia | Non-Hodgkin lymphoma | 3512.169 | 0.183798 |
| Oceania | Non-Hodgkin lymphoma | 19.175 | 0.162307 |
| Southeast Asia | Non-Hodgkin lymphoma | 2052.019 | 0.257195 |
| Central Sub-Saharan Africa | Non-Hodgkin lymphoma | 206.1225 | 0.298135 |
| Eastern Sub-Saharan Africa | Non-Hodgkin lymphoma | 608.4926 | 0.223675 |
| Southern Sub-Saharan Africa | Non-Hodgkin lymphoma | 504.1811 | 0.495368 |
| Western Sub-Saharan Africa | Non-Hodgkin lymphoma | 688.2357 | 0.278872 |
| Global | Multiple myeloma | 1420.119 | 0.018554 |
| Low SDI | Multiple myeloma | 130.6734 | 0.016275 |
| Low-middle SDI | Multiple myeloma | 282.2922 | 0.017074 |
| Middle SDI | Multiple myeloma | 473.2279 | 0.01672 |
| High-middle SDI | Multiple myeloma | 349.5118 | 0.020955 |
| High SDI | Multiple myeloma | 183.5647 | 0.026423 |
| Central Asia | Multiple myeloma | 20.05653 | 0.015728 |
| Central Europe | Multiple myeloma | 23.72505 | 0.020558 |
| Eastern Europe | Multiple myeloma | 48.87445 | 0.018993 |
| Australasia | Multiple myeloma | 5.998712 | 0.034363 |
| High-income Asia Pacific | Multiple myeloma | 20.06749 | 0.018784 |
| High-income North America | Multiple myeloma | 71.37372 | 0.027627 |
| Southern Latin America | Multiple myeloma | 12.38137 | 0.015334 |
| Western Europe | Multiple myeloma | 77.72616 | 0.026756 |
| Andean Latin America | Multiple myeloma | 12.83498 | 0.013554 |
| Caribbean | Multiple myeloma | 11.61726 | 0.02022 |
| Central Latin America | Multiple myeloma | 44.33495 | 0.012744 |
| Tropical Latin America | Multiple myeloma | 50.2737 | 0.020888 |
| North Africa and Middle East | Multiple myeloma | 106.3302 | 0.014666 |
| South Asia | Multiple myeloma | 291.8648 | 0.019154 |
| East Asia | Multiple myeloma | 425.7756 | 0.022282 |
| Oceania | Multiple myeloma | 2.568526 | 0.021741 |
| Southeast Asia | Multiple myeloma | 74.30085 | 0.009313 |
| Central Sub-Saharan Africa | Multiple myeloma | 10.7135 | 0.015496 |
| Eastern Sub-Saharan Africa | Multiple myeloma | 53.59764 | 0.019702 |
| Southern Sub-Saharan Africa | Multiple myeloma | 26.93023 | 0.026459 |
| Western Sub-Saharan Africa | Multiple myeloma | 28.77358 | 0.011659 |
| Global | Acute lymphoid leukemia | 11726.39 | 0.153206 |
| Low SDI | Acute lymphoid leukemia | 928.929 | 0.115697 |
| Low-middle SDI | Acute lymphoid leukemia | 2150.764 | 0.130088 |
| Middle SDI | Acute lymphoid leukemia | 4789.751 | 0.169233 |
| High-middle SDI | Acute lymphoid leukemia | 2764.552 | 0.165745 |
| High SDI | Acute lymphoid leukemia | 1086.483 | 0.156394 |
| Central Asia | Acute lymphoid leukemia | 201.2934 | 0.157846 |
| Central Europe | Acute lymphoid leukemia | 175.3186 | 0.151914 |
| Eastern Europe | Acute lymphoid leukemia | 489.1404 | 0.190084 |
| Australasia | Acute lymphoid leukemia | 30.92025 | 0.177124 |
| High-income Asia Pacific | Acute lymphoid leukemia | 193.755 | 0.181359 |
| High-income North America | Acute lymphoid leukemia | 442.7635 | 0.171386 |
| Southern Latin America | Acute lymphoid leukemia | 157.2223 | 0.194719 |
| Western Europe | Acute lymphoid leukemia | 445.9468 | 0.153512 |
| Andean Latin America | Acute lymphoid leukemia | 211.5676 | 0.223415 |
| Caribbean | Acute lymphoid leukemia | 67.41538 | 0.117335 |
| Central Latin America | Acute lymphoid leukemia | 1127.913 | 0.324214 |
| Tropical Latin America | Acute lymphoid leukemia | 442.76 | 0.18396 |
| North Africa and Middle East | Acute lymphoid leukemia | 1052.772 | 0.145204 |
| South Asia | Acute lymphoid leukemia | 1878.548 | 0.123279 |
| East Asia | Acute lymphoid leukemia | 3142.657 | 0.164461 |
| Oceania | Acute lymphoid leukemia | 10.38493 | 0.087903 |
| Southeast Asia | Acute lymphoid leukemia | 990.2144 | 0.124111 |
| Central Sub-Saharan Africa | Acute lymphoid leukemia | 57.78171 | 0.083575 |
| Eastern Sub-Saharan Africa | Acute lymphoid leukemia | 333.351 | 0.122536 |
| Southern Sub-Saharan Africa | Acute lymphoid leukemia | 61.15519 | 0.060086 |
| Western Sub-Saharan Africa | Acute lymphoid leukemia | 213.5069 | 0.086513 |
| Global | Acute myeloid leukemia | 11166.42 | 0.14589 |
| Low SDI | Acute myeloid leukemia | 898.9266 | 0.111961 |
| Low-middle SDI | Acute myeloid leukemia | 2137.246 | 0.129271 |
| Middle SDI | Acute myeloid leukemia | 3724.08 | 0.131581 |
| High-middle SDI | Acute myeloid leukemia | 2630.423 | 0.157703 |
| High SDI | Acute myeloid leukemia | 1767.215 | 0.254383 |
| Central Asia | Acute myeloid leukemia | 236.4271 | 0.185397 |
| Central Europe | Acute myeloid leukemia | 261.4397 | 0.226538 |
| Eastern Europe | Acute myeloid leukemia | 364.959 | 0.141826 |
| Australasia | Acute myeloid leukemia | 51.91493 | 0.29739 |
| High-income Asia Pacific | Acute myeloid leukemia | 343.1545 | 0.321201 |
| High-income North America | Acute myeloid leukemia | 653.8119 | 0.253079 |
| Southern Latin America | Acute myeloid leukemia | 152.8137 | 0.189259 |
| Western Europe | Acute myeloid leukemia | 721.78 | 0.248464 |
| Andean Latin America | Acute myeloid leukemia | 131.1381 | 0.138481 |
| Caribbean | Acute myeloid leukemia | 90.3591 | 0.157268 |
| Central Latin America | Acute myeloid leukemia | 608.6327 | 0.174949 |
| Tropical Latin America | Acute myeloid leukemia | 568.5818 | 0.236237 |
| North Africa and Middle East | Acute myeloid leukemia | 1397.08 | 0.192693 |
| South Asia | Acute myeloid leukemia | 2114.286 | 0.138749 |
| East Asia | Acute myeloid leukemia | 1610.615 | 0.084287 |
| Oceania | Acute myeloid leukemia | 31.96861 | 0.270599 |
| Southeast Asia | Acute myeloid leukemia | 1151.264 | 0.144297 |
| Central Sub-Saharan Africa | Acute myeloid leukemia | 55.28696 | 0.079967 |
| Eastern Sub-Saharan Africa | Acute myeloid leukemia | 225.3174 | 0.082824 |
| Southern Sub-Saharan Africa | Acute myeloid leukemia | 76.57735 | 0.075239 |
| Western Sub-Saharan Africa | Acute myeloid leukemia | 319.0143 | 0.129264 |
| Global | Chronic lymphoid leukemia | 895.3832 | 0.011698 |
| Low SDI | Chronic lymphoid leukemia | 46.82819 | 0.005832 |
| Low-middle SDI | Chronic lymphoid leukemia | 136.8806 | 0.008279 |
| Middle SDI | Chronic lymphoid leukemia | 416.3976 | 0.014712 |
| High-middle SDI | Chronic lymphoid leukemia | 241.7096 | 0.014491 |
| High SDI | Chronic lymphoid leukemia | 53.29048 | 0.007671 |
| Central Asia | Chronic lymphoid leukemia | 14.64782 | 0.011486 |
| Central Europe | Chronic lymphoid leukemia | 15.60154 | 0.013519 |
| Eastern Europe | Chronic lymphoid leukemia | 32.36526 | 0.012577 |
| Australasia | Chronic lymphoid leukemia | 0.995016 | 0.0057 |
| High-income Asia Pacific | Chronic lymphoid leukemia | 6.541326 | 0.006123 |
| High-income North America | Chronic lymphoid leukemia | 13.85661 | 0.005364 |
| Southern Latin America | Chronic lymphoid leukemia | 3.203386 | 0.003967 |
| Western Europe | Chronic lymphoid leukemia | 27.34592 | 0.009414 |
| Andean Latin America | Chronic lymphoid leukemia | 7.280539 | 0.007688 |
| Caribbean | Chronic lymphoid leukemia | 3.123075 | 0.005436 |
| Central Latin America | Chronic lymphoid leukemia | 17.50246 | 0.005031 |
| Tropical Latin America | Chronic lymphoid leukemia | 9.349198 | 0.003884 |
| North Africa and Middle East | Chronic lymphoid leukemia | 69.28098 | 0.009556 |
| South Asia | Chronic lymphoid leukemia | 92.82442 | 0.006092 |
| East Asia | Chronic lymphoid leukemia | 496.9586 | 0.026007 |
| Oceania | Chronic lymphoid leukemia | 0.438597 | 0.003713 |
| Southeast Asia | Chronic lymphoid leukemia | 33.87233 | 0.004245 |
| Central Sub-Saharan Africa | Chronic lymphoid leukemia | 5.208815 | 0.007534 |
| Eastern Sub-Saharan Africa | Chronic lymphoid leukemia | 9.993321 | 0.003673 |
| Southern Sub-Saharan Africa | Chronic lymphoid leukemia | 21.44582 | 0.021071 |
| Western Sub-Saharan Africa | Chronic lymphoid leukemia | 13.54822 | 0.00549 |
| Global | Chronic myeloid leukemia | 4940.246 | 0.064545 |
| Low SDI | Chronic myeloid leukemia | 1061.487 | 0.132207 |
| Low-middle SDI | Chronic myeloid leukemia | 1656.294 | 0.10018 |
| Middle SDI | Chronic myeloid leukemia | 1305.475 | 0.046126 |
| High-middle SDI | Chronic myeloid leukemia | 630.269 | 0.037787 |
| High SDI | Chronic myeloid leukemia | 283.92 | 0.040869 |
| Central Asia | Chronic myeloid leukemia | 50.21622 | 0.039378 |
| Central Europe | Chronic myeloid leukemia | 47.29525 | 0.040981 |
| Eastern Europe | Chronic myeloid leukemia | 150.1703 | 0.058358 |
| Australasia | Chronic myeloid leukemia | 8.258412 | 0.047308 |
| High-income Asia Pacific | Chronic myeloid leukemia | 50.80866 | 0.047558 |
| High-income North America | Chronic myeloid leukemia | 89.18297 | 0.034521 |
| Southern Latin America | Chronic myeloid leukemia | 29.07855 | 0.036014 |
| Western Europe | Chronic myeloid leukemia | 99.93386 | 0.034401 |
| Andean Latin America | Chronic myeloid leukemia | 32.49223 | 0.034312 |
| Caribbean | Chronic myeloid leukemia | 29.80901 | 0.051882 |
| Central Latin America | Chronic myeloid leukemia | 127.305 | 0.036593 |
| Tropical Latin America | Chronic myeloid leukemia | 110.832 | 0.046049 |
| North Africa and Middle East | Chronic myeloid leukemia | 538.24 | 0.074237 |
| South Asia | Chronic myeloid leukemia | 2273.593 | 0.149204 |
| East Asia | Chronic myeloid leukemia | 280.2456 | 0.014666 |
| Oceania | Chronic myeloid leukemia | 7.518394 | 0.06364 |
| Southeast Asia | Chronic myeloid leukemia | 310.2418 | 0.038885 |
| Central Sub-Saharan Africa | Chronic myeloid leukemia | 50.87295 | 0.073582 |
| Eastern Sub-Saharan Africa | Chronic myeloid leukemia | 423.419 | 0.155644 |
| Southern Sub-Saharan Africa | Chronic myeloid leukemia | 14.64824 | 0.014392 |
| Western Sub-Saharan Africa | Chronic myeloid leukemia | 216.0838 | 0.087557 |
| Global | Other leukemia | 19001.21 | 0.248252 |
| Low SDI | Other leukemia | 1376.4 | 0.17143 |
| Low-middle SDI | Other leukemia | 3384.131 | 0.204688 |
| Middle SDI | Other leukemia | 8918.912 | 0.315127 |
| High-middle SDI | Other leukemia | 4472.541 | 0.268145 |
| High SDI | Other leukemia | 836.9101 | 0.120469 |
| Central Asia | Other leukemia | 313.9508 | 0.246188 |
| Central Europe | Other leukemia | 122.4604 | 0.106112 |
| Eastern Europe | Other leukemia | 221.0669 | 0.085909 |
| Australasia | Other leukemia | 8.042646 | 0.046072 |
| High-income Asia Pacific | Other leukemia | 112.4629 | 0.105268 |
| High-income North America | Other leukemia | 267.5871 | 0.103578 |
| Southern Latin America | Other leukemia | 132.6909 | 0.164337 |
| Western Europe | Other leukemia | 287.1813 | 0.098859 |
| Andean Latin America | Other leukemia | 247.6126 | 0.261478 |
| Caribbean | Other leukemia | 126.9999 | 0.221041 |
| Central Latin America | Other leukemia | 570.5139 | 0.163992 |
| Tropical Latin America | Other leukemia | 316.6282 | 0.131554 |
| North Africa and Middle East | Other leukemia | 1491.315 | 0.205691 |
| South Asia | Other leukemia | 1368.69 | 0.08982 |
| East Asia | Other leukemia | 9175.828 | 0.480188 |
| Oceania | Other leukemia | 37.69321 | 0.319054 |
| Southeast Asia | Other leukemia | 2915.71 | 0.365448 |
| Central Sub-Saharan Africa | Other leukemia | 217.0811 | 0.313985 |
| Eastern Sub-Saharan Africa | Other leukemia | 378.0587 | 0.13897 |
| Southern Sub-Saharan Africa | Other leukemia | 230.7421 | 0.226709 |
| Western Sub-Saharan Africa | Other leukemia | 458.8902 | 0.185942 |

**Table S 3** The proportional incidence rate of AYA (Age subgroup analysis)

| age_name | cause_name | n | percentage |
| --- | --- | --- | --- |
| 15-19 years | Hodgkin lymphoma | 4957.653 | 0.14826 |
| 20-24 years | Hodgkin lymphoma | 7573.796 | 0.216231 |
| 25-29 years | Hodgkin lymphoma | 7810.137 | 0.213365 |
| 30-34 years | Hodgkin lymphoma | 7170.407 | 0.180573 |
| 35-39 years | Hodgkin lymphoma | 5875.837 | 0.130074 |
| 15-19 years | Non-Hodgkin lymphoma | 10399.85 | 0.31101 |
| 20-24 years | Non-Hodgkin lymphoma | 8343.806 | 0.238214 |
| 25-29 years | Non-Hodgkin lymphoma | 9228.967 | 0.252126 |
| 30-34 years | Non-Hodgkin lymphoma | 11199.49 | 0.282037 |
| 35-39 years | Non-Hodgkin lymphoma | 13254.79 | 0.293423 |
| 15-19 years | Multiple myeloma | 0 | 0 |
| 20-24 years | Multiple myeloma | 367.5197 | 0.010493 |
| 25-29 years | Multiple myeloma | 429.5443 | 0.011735 |
| 30-34 years | Multiple myeloma | 767.8882 | 0.019338 |
| 35-39 years | Multiple myeloma | 1365.859 | 0.030236 |
| 15-19 years | Acute lymphoid leukemia | 8348.989 | 0.249678 |
| 20-24 years | Acute lymphoid leukemia | 7679.245 | 0.219241 |
| 25-29 years | Acute lymphoid leukemia | 6733.699 | 0.183958 |
| 30-34 years | Acute lymphoid leukemia | 7551.935 | 0.190181 |
| 35-39 years | Acute lymphoid leukemia | 8432.672 | 0.186675 |
| 15-19 years | Acute myeloid leukemia | 4575.752 | 0.136839 |
| 20-24 years | Acute myeloid leukemia | 3828.264 | 0.109296 |
| 25-29 years | Acute myeloid leukemia | 3883.338 | 0.106089 |
| 30-34 years | Acute myeloid leukemia | 3759.822 | 0.094684 |
| 35-39 years | Acute myeloid leukemia | 4135.544 | 0.091549 |
| 15-19 years | Chronic lymphoid leukemia | 0 | 0 |
| 20-24 years | Chronic lymphoid leukemia | 946.1484 | 0.027012 |
| 25-29 years | Chronic lymphoid leukemia | 953.0646 | 0.026037 |
| 30-34 years | Chronic lymphoid leukemia | 1122.322 | 0.028263 |
| 35-39 years | Chronic lymphoid leukemia | 1241.383 | 0.027481 |
| 15-19 years | Chronic myeloid leukemia | 892.5125 | 0.026691 |
| 20-24 years | Chronic myeloid leukemia | 1389.354 | 0.039666 |
| 25-29 years | Chronic myeloid leukemia | 2043.315 | 0.055821 |
| 30-34 years | Chronic myeloid leukemia | 2264.574 | 0.057029 |
| 35-39 years | Chronic myeloid leukemia | 2613.473 | 0.057855 |
| 15-19 years | Other leukemia | 4264.2 | 0.127522 |
| 20-24 years | Other leukemia | 4898.348 | 0.139847 |
| 25-29 years | Other leukemia | 5522.525 | 0.15087 |
| 30-34 years | Other leukemia | 5872.844 | 0.147896 |
| 35-39 years | Other leukemia | 8253.385 | 0.182706 |

**Table S 4** The proportional death rate of AYA (Age subgroup analysis)

| age_name | cause_name | n | percentage |
| --- | --- | --- | --- |
| 15-19 years | Hodgkin lymphoma | 1095.858 | 0.085987 |
| 20-24 years | Hodgkin lymphoma | 1592.739 | 0.113787 |
| 25-29 years | Hodgkin lymphoma | 1924.49 | 0.12969 |
| 30-34 years | Hodgkin lymphoma | 1808.825 | 0.11219 |
| 35-39 years | Hodgkin lymphoma | 1671.401 | 0.092998 |
| 15-19 years | Non-Hodgkin lymphoma | 2826.693 | 0.221797 |
| 20-24 years | Non-Hodgkin lymphoma | 3673.124 | 0.262412 |
| 25-29 years | Non-Hodgkin lymphoma | 3972.617 | 0.267712 |
| 30-34 years | Non-Hodgkin lymphoma | 4674.483 | 0.289929 |
| 35-39 years | Non-Hodgkin lymphoma | 5650.444 | 0.314394 |
| 15-19 years | Multiple myeloma | 0 | 0 |
| 20-24 years | Multiple myeloma | 181.6502 | 0.012977 |
| 25-29 years | Multiple myeloma | 223.1717 | 0.015039 |
| 30-34 years | Multiple myeloma | 449.5983 | 0.027886 |
| 35-39 years | Multiple myeloma | 825.8471 | 0.045951 |
| 15-19 years | Acute lymphoid leukemia | 3568.881 | 0.280033 |
| 20-24 years | Acute lymphoid leukemia | 2514.237 | 0.17962 |
| 25-29 years | Acute lymphoid leukemia | 2081.306 | 0.140258 |
| 30-34 years | Acute lymphoid leukemia | 1796.689 | 0.111437 |
| 35-39 years | Acute lymphoid leukemia | 1735.382 | 0.096558 |
| 15-19 years | Acute myeloid leukemia | 2297.393 | 0.180265 |
| 20-24 years | Acute myeloid leukemia | 2124.327 | 0.151764 |
| 25-29 years | Acute myeloid leukemia | 2343.16 | 0.157904 |
| 30-34 years | Acute myeloid leukemia | 2512.364 | 0.155826 |
| 35-39 years | Acute myeloid leukemia | 2888.94 | 0.160742 |
| 15-19 years | Chronic lymphoid leukemia | 0 | 0 |
| 20-24 years | Chronic lymphoid leukemia | 208.5294 | 0.014898 |
| 25-29 years | Chronic lymphoid leukemia | 214.4331 | 0.01445 |
| 30-34 years | Chronic lymphoid leukemia | 263.1367 | 0.016321 |
| 35-39 years | Chronic lymphoid leukemia | 323.6392 | 0.018007 |
| 15-19 years | Chronic myeloid leukemia | 414.2889 | 0.032507 |
| 20-24 years | Chronic myeloid leukemia | 701.7406 | 0.050133 |
| 25-29 years | Chronic myeloid leukemia | 1079.564 | 0.072751 |
| 30-34 years | Chronic myeloid leukemia | 1256.878 | 0.077956 |
| 35-39 years | Chronic myeloid leukemia | 1503.173 | 0.083637 |
| 15-19 years | Other leukemia | 2541.405 | 0.199412 |
| 20-24 years | Other leukemia | 3001.22 | 0.21441 |
| 25-29 years | Other leukemia | 3000.429 | 0.202197 |
| 30-34 years | Other leukemia | 3360.886 | 0.208455 |
| 35-39 years | Other leukemia | 3373.65 | 0.187712 |

**Table S 5** Changes in the ASIR (per 100000) of hematological tumors from 1990 to 2019 by gender, as well as the predicted changes from 2020 to 2044.

|  | 1990-1994 | 1995-1999 | 2000-2004 | 2005-2009 | 2010-2014 | 2015-2019 | 2020-2024 | 2025-2029 | 2030-2034 | 2035-2039 | 2040-2044 |
| --- | --- | --- | --- | --- | --- | --- | --- | --- | --- | --- | --- |
| Non-Hodgkin lymphoma | | | | | | | | | | | |
| Male | 1.890 | 1.922 | 1.877 | 1.964 | 2.055 | 2.132 | 2.162 | 2.148 | 2.117 | 2.076 | 2.049 |
| Female | 1.116 | 1.211 | 1.189 | 1.183 | 1.231 | 1.262 | 1.279 | 1.273 | 1.262 | 1.249 | 1.237 |
| Both | 1.508 | 1.571 | 1.536 | 1.577 | 1.647 | 1.702 | 1.726 | 1.718 | 1.698 | 1.673 | 1.654 |
| Hodgkin lymphoma | | | | | | | | | | | |
| Male | 1.366 | 1.294 | 1.226 | 1.223 | 1.193 | 1.183 | 1.164 | 1.150 | 1.140 | 1.131 | 1.121 |
| Female | 1.076 | 1.066 | 1.036 | 1.022 | 1.030 | 1.029 | 1.039 | 1.054 | 1.077 | 1.098 | 1.113 |
| Both | 1.223 | 1.181 | 1.132 | 1.123 | 1.112 | 1.106 | 1.100 | 1.097 | 1.100 | 1.102 | 1.103 |
| Leukemia | | | | | | | | | | | |
| Male | 3.895 | 3.847 | 3.837 | 3.718 | 3.602 | 3.662 | 3.424 | 3.146 | 2.839 | 2.602 | 2.397 |
| Female | 3.443 | 3.643 | 3.483 | 3.144 | 3.047 | 2.990 | 2.707 | 2.421 | 2.106 | 1.861 | 1.648 |
| Both | 3.672 | 3.747 | 3.662 | 3.435 | 3.328 | 3.330 | 3.069 | 2.785 | 2.468 | 2.222 | 2.007 |
| Multiple myeloma | | | | | | | | | | | |
| Male | 0.087 | 0.099 | 0.102 | 0.103 | 0.109 | 0.117 | 0.125 | 0.132 | 0.135 | 0.136 | 0.137 |
| Female | 0.060 | 0.076 | 0.074 | 0.068 | 0.071 | 0.072 | 0.072 | 0.073 | 0.072 | 0.071 | 0.071 |
| Both | 0.074 | 0.088 | 0.088 | 0.086 | 0.090 | 0.095 | 0.099 | 0.103 | 0.103 | 0.103 | 0.103 |

**Table S 6** Changes in the ASDR (per 100000) of hematological tumors from 1990 to 2019 by gender, as well as the predicted changes from 2020 to 2044.

|  | 1990-1994 | 1995-1999 | 2000-2004 | 2005-2009 | 2010-2014 | 2015-2019 | 2020-2024 | 2025-2029 | 2030-2034 | 2035-2039 | 2040-2044 |
| --- | --- | --- | --- | --- | --- | --- | --- | --- | --- | --- | --- |
| Non-Hodgkin lymphoma | | | | | | | | | | | |
| Male | 0.886 | 0.882 | 0.852 | 0.856 | 0.857 | 0.855 | 0.830 | 0.802 | 0.777 | 0.754 | 0.735 |
| Female | 0.542 | 0.568 | 0.542 | 0.513 | 0.507 | 0.504 | 0.491 | 0.476 | 0.464 | 0.454 | 0.444 |
| Both | 0.716 | 0.727 | 0.698 | 0.686 | 0.684 | 0.682 | 0.663 | 0.642 | 0.623 | 0.607 | 0.593 |
| Hodgkin lymphoma | | | | | | | | | | | |
| Male | 0.489 | 0.446 | 0.398 | 0.367 | 0.341 | 0.323 | 0.305 | 0.296 | 0.294 | 0.296 | 0.293 |
| Female | 0.314 | 0.293 | 0.263 | 0.238 | 0.227 | 0.220 | 0.213 | 0.212 | 0.216 | 0.223 | 0.226 |
| Both | 0.403 | 0.371 | 0.331 | 0.303 | 0.284 | 0.272 | 0.259 | 0.253 | 0.254 | 0.257 | 0.256 |
| Leukemia | | | | | | | | | | | |
| Male | 2.403 | 2.265 | 2.156 | 1.981 | 1.813 | 1.741 | 1.586 | 1.443 | 1.319 | 1.239 | 1.165 |
| Female | 2.014 | 2.036 | 1.847 | 1.559 | 1.407 | 1.297 | 1.142 | 1.010 | 0.887 | 0.806 | 0.730 |
| Both | 2.211 | 2.152 | 2.003 | 1.772 | 1.612 | 1.522 | 1.366 | 1.228 | 1.103 | 1.020 | 0.944 |
| Multiple myeloma | | | | | | | | | | | |
| Male | 0.057 | 0.062 | 0.063 | 0.062 | 0.064 | 0.067 | 0.070 | 0.072 | 0.073 | 0.073 | 0.073 |
| Female | 0.039 | 0.048 | 0.046 | 0.040 | 0.041 | 0.041 | 0.040 | 0.039 | 0.038 | 0.038 | 0.038 |
| Both | 0.048 | 0.055 | 0.054 | 0.051 | 0.052 | 0.054 | 0.055 | 0.056 | 0.056 | 0.055 | 0.055 |

**Table S 7** Changes in the incidence rates (per 100000) of hematological tumors among different age groups from 1990 to 2019, as well as the predicted changes in the incidence rates from 2020 to 2044.

|  | | 1990-1994 | 1995-1999 | 2000-2004 | 2005-2009 | 2010-2014 | 2015-2019 | 2020-2024 | 2025-2029 | 2030-2034 | 2035-2039 | 2040-2044 |
| --- | --- | --- | --- | --- | --- | --- | --- | --- | --- | --- | --- | --- |
| Non-Hodgkin lymphoma | |  |  |  |  |  |  |  |  |  |  |  |
|  | 15-19y | 1.554 | 1.595 | 1.577 | 1.606 | 1.629 | 1.630 | 1.624 | 1.583 | 1.609 | 1.622 | 1.635 |
|  | 20-24y | 1.077 | 1.174 | 1.207 | 1.264 | 1.321 | 1.335 | 1.333 | 1.312 | 1.266 | 1.277 | 1.288 |
|  | 25-29y | 1.218 | 1.297 | 1.305 | 1.346 | 1.442 | 1.482 | 1.487 | 1.486 | 1.450 | 1.389 | 1.401 |
|  | 30-34y | 1.587 | 1.618 | 1.548 | 1.575 | 1.683 | 1.790 | 1.824 | 1.813 | 1.797 | 1.742 | 1.671 |
|  | 35-39y | 2.189 | 2.257 | 2.115 | 2.165 | 2.239 | 2.366 | 2.467 | 2.508 | 2.476 | 2.437 | 2.366 |
| Hodgkin lymphoma | |  |  |  |  |  |  |  |  |  |  |  |
|  | 15-19y | 0.851 | 0.846 | 0.805 | 0.789 | 0.785 | 0.786 | 0.792 | 0.790 | 0.792 | 0.791 | 0.791 |
|  | 20-24y | 1.284 | 1.297 | 1.299 | 1.267 | 1.238 | 1.239 | 1.251 | 1.250 | 1.247 | 1.251 | 1.251 |
|  | 25-29y | 1.374 | 1.320 | 1.304 | 1.336 | 1.298 | 1.269 | 1.269 | 1.283 | 1.283 | 1.281 | 1.285 |
|  | 30-34y | 1.384 | 1.247 | 1.171 | 1.181 | 1.205 | 1.190 | 1.151 | 1.150 | 1.164 | 1.164 | 1.162 |
|  | 35-39y | 1.263 | 1.233 | 1.102 | 1.066 | 1.058 | 1.074 | 1.058 | 1.031 | 1.031 | 1.044 | 1.044 |
| Leukemia | |  |  |  |  |  |  |  |  |  |  |  |
|  | 15-19y | 4.073 | 3.843 | 3.674 | 3.340 | 3.048 | 2.911 | 2.423 | 2.041 | 1.721 | 1.703 | 1.684 |
|  | 20-24y | 3.432 | 3.462 | 3.304 | 3.141 | 3.118 | 3.073 | 2.664 | 2.255 | 1.916 | 1.630 | 1.612 |
|  | 25-29y | 3.298 | 3.537 | 3.322 | 3.054 | 3.124 | 3.137 | 2.855 | 2.596 | 2.219 | 1.904 | 1.620 |
|  | 30-34y | 3.154 | 3.474 | 3.407 | 3.155 | 3.137 | 3.271 | 3.168 | 2.902 | 2.666 | 2.304 | 1.980 |
|  | 35-39y | 4.434 | 4.486 | 4.719 | 4.616 | 4.345 | 4.421 | 4.460 | 4.390 | 4.081 | 3.806 | 3.323 |
| Multiple myeloma | |  |  |  |  |  |  |  |  |  |  |  |
|  | 15-19y | 0.000 | 0.000 | 0.000 | 0.000 | 0.000 | 0.000 | 0.000 | 0.000 | 0.000 | 0.000 | 0.000 |
|  | 20-24y | 0.040 | 0.053 | 0.053 | 0.052 | 0.060 | 0.061 | 0.062 | 0.062 | 0.062 | 0.062 | 0.062 |
|  | 25-29y | 0.046 | 0.062 | 0.061 | 0.057 | 0.066 | 0.071 | 0.072 | 0.073 | 0.073 | 0.073 | 0.073 |
|  | 30-34y | 0.095 | 0.115 | 0.116 | 0.112 | 0.116 | 0.123 | 0.134 | 0.135 | 0.136 | 0.136 | 0.136 |
|  | 35-39y | 0.213 | 0.235 | 0.238 | 0.235 | 0.235 | 0.246 | 0.254 | 0.273 | 0.275 | 0.275 | 0.276 |

**Table S 8** Changes in the mortality rate (per 100000) of hematological tumors among different age groups from 1990 to 2019, as well as the predicted changes in the incidence rates from 2020 to 2044.

|  |  | 1990-1994 | | 1995-1999 | | 2000-2004 | | 2005-2009 | | 2010-2014 | | 2015-2019 | | 2020-2024 | 2025-2029 | 2030-2034 | 2035-2039 | 2040-2044 |
| --- | --- | --- | --- | --- | --- | --- | --- | --- | --- | --- | --- | --- | --- | --- | --- | --- | --- | --- |
| Non-Hodgkin lymphoma | |  |  | |  | |  | |  | |  | |  | |  |  |  |  |
|  | 15-19y | 0.525 | | 0.516 | | 0.490 | | 0.473 | | 0.466 | | 0.451 | | 0.428 | 0.402 | 0.399 | 0.398 | 0.396 |
|  | 20-24y | 0.583 | | 0.619 | | 0.613 | | 0.606 | | 0.600 | | 0.594 | | 0.570 | 0.542 | 0.512 | 0.511 | 0.509 |
|  | 25-29y | 0.643 | | 0.674 | | 0.667 | | 0.652 | | 0.654 | | 0.641 | | 0.630 | 0.612 | 0.584 | 0.554 | 0.552 |
|  | 30-34y | 0.798 | | 0.801 | | 0.763 | | 0.749 | | 0.753 | | 0.762 | | 0.740 | 0.727 | 0.710 | 0.680 | 0.646 |
|  | 35-39y | 1.095 | | 1.087 | | 1.018 | | 1.011 | | 1.005 | | 1.024 | | 1.009 | 0.990 | 0.977 | 0.957 | 0.920 |
| Hodgkin lymphoma | |  |  |  |  |  |  |  |  |  |  |  |  |  |  |  |  |  |
|  | 15-19y | 0.264 | | 0.242 | | 0.212 | | 0.192 | | 0.186 | | 0.179 | | 0.175 | 0.167 | 0.163 | 0.159 | 0.155 |
|  | 20-24y | 0.364 | | 0.350 | | 0.320 | | 0.288 | | 0.266 | | 0.263 | | 0.258 | 0.251 | 0.246 | 0.248 | 0.242 |
|  | 25-29y | 0.445 | | 0.420 | | 0.392 | | 0.367 | | 0.336 | | 0.314 | | 0.308 | 0.309 | 0.308 | 0.309 | 0.310 |
|  | 30-34y | 0.474 | | 0.417 | | 0.371 | | 0.346 | | 0.327 | | 0.307 | | 0.282 | 0.282 | 0.289 | 0.295 | 0.296 |
|  | 35-39y | 0.493 | | 0.447 | | 0.378 | | 0.339 | | 0.320 | | 0.312 | | 0.285 | 0.268 | 0.274 | 0.287 | 0.293 |
| Leukemia |  |  |  |  |  |  |  |  |  |  |  |  |  |  |  |  |  |  |
|  | 15-19y | 2.339 | | 2.149 | | 2.020 | | 1.784 | | 1.566 | | 1.444 | | 1.253 | 1.079 | 0.918 | 0.896 | 0.874 |
|  | 20-24y | 2.064 | | 1.991 | | 1.810 | | 1.642 | | 1.527 | | 1.427 | | 1.253 | 1.091 | 0.959 | 0.832 | 0.812 |
|  | 25-29y | 1.997 | | 2.051 | | 1.846 | | 1.605 | | 1.537 | | 1.452 | | 1.277 | 1.172 | 1.043 | 0.938 | 0.814 |
|  | 30-34y | 2.061 | | 2.143 | | 1.984 | | 1.715 | | 1.574 | | 1.515 | | 1.398 | 1.258 | 1.182 | 1.077 | 0.969 |
|  | 35-39y | 2.626 | | 2.465 | | 2.402 | | 2.158 | | 1.894 | | 1.811 | | 1.703 | 1.600 | 1.476 | 1.420 | 1.299 |
| Multiple myeloma | |  |  |  |  |  |  |  |  |  |  |  |  |  |  |  |  |  |
|  | 15-19y | 0.000 | | 0.000 | | 0.000 | | 0.000 | | 0.000 | | 0.000 | | 0.000 | 0.000 | 0.000 | 0.000 | 0.000 |
|  | 20-24y | 0.023 | | 0.030 | | 0.029 | | 0.027 | | 0.030 | | 0.030 | | 0.030 | 0.029 | 0.029 | 0.029 | 0.029 |
|  | 25-29y | 0.028 | | 0.037 | | 0.035 | | 0.032 | | 0.035 | | 0.037 | | 0.037 | 0.036 | 0.036 | 0.036 | 0.035 |
|  | 30-34y | 0.062 | | 0.074 | | 0.073 | | 0.068 | | 0.069 | | 0.072 | | 0.076 | 0.075 | 0.074 | 0.074 | 0.074 |
|  | 35-39y | 0.142 | | 0.152 | | 0.150 | | 0.144 | | 0.143 | | 0.149 | | 0.150 | 0.158 | 0.156 | 0.156 | 0.155 |

**Table S 9** Changes in the ASIR (per 100000) of leukemia from 1990 to 2019, as well as the predicted changes in the ASIR from 2020 to 2044.

| disease | 1990-1994 | 1995-1999 | 2000-2004 | 2005-2009 | 2010-2014 | 2015-2019 | 2020-2024 | 2025-2029 | 2030-2034 | 2035-2039 | 2040-2044 |
| --- | --- | --- | --- | --- | --- | --- | --- | --- | --- | --- | --- |
| Acute lymphoid leukemia | 0.810 | 0.887 | 0.949 | 1.024 | 1.145 | 1.252 | 1.288 | 1.276 | 1.228 | 1.172 | 1.146 |
| Chronic lymphoid leukemia | 0.068 | 0.075 | 0.081 | 0.093 | 0.117 | 0.133 | 0.154 | 0.169 | 0.176 | 0.178 | 0.181 |
| Acute myeloid leukemia | 0.649 | 0.663 | 0.660 | 0.663 | 0.672 | 0.674 | 0.663 | 0.650 | 0.634 | 0.616 | 0.605 |
| Chronic myeloid leukemia | 0.377 | 0.395 | 0.359 | 0.322 | 0.302 | 0.296 | 0.278 | 0.260 | 0.242 | 0.226 | 0.205 |
| Other leukemia | 1.767 | 1.726 | 1.613 | 1.333 | 1.091 | 0.975 | 0.781 | 0.629 | 0.501 | 0.426 | 0.364 |

**Table S 10** Changes in the ASDR (per 100000) of leukemia from 1990 to 2019, as well as the predicted changes in the ASDR from 2020 to 2044.

| disease | 1990-1994 | 1995-1999 | 2000-2004 | 2005-2009 | 2010-2014 | 2015-2019 | 2020-2024 | 2025-2029 | 2030-2034 | 2035-2039 | 2040-2044 |
| --- | --- | --- | --- | --- | --- | --- | --- | --- | --- | --- | --- |
| Acute lymphoid leukemia | 0.437 | 0.425 | 0.412 | 0.411 | 0.413 | 0.401 | 0.384 | 0.361 | 0.335 | 0.317 | 0.303 |
| Chronic lymphoid leukemia | 0.033 | 0.034 | 0.033 | 0.031 | 0.032 | 0.033 | 0.034 | 0.034 | 0.033 | 0.033 | 0.033 |
| Acute myeloid leukemia | 0.408 | 0.407 | 0.399 | 0.397 | 0.401 | 0.404 | 0.395 | 0.386 | 0.374 | 0.361 | 0.351 |
| Chronic myeloid leukemia | 0.243 | 0.232 | 0.206 | 0.184 | 0.170 | 0.161 | 0.148 | 0.136 | 0.127 | 0.119 | 0.107 |
| Other leukemia | 1.091 | 1.054 | 0.954 | 0.749 | 0.596 | 0.523 | 0.409 | 0.325 | 0.260 | 0.223 | 0.193 |


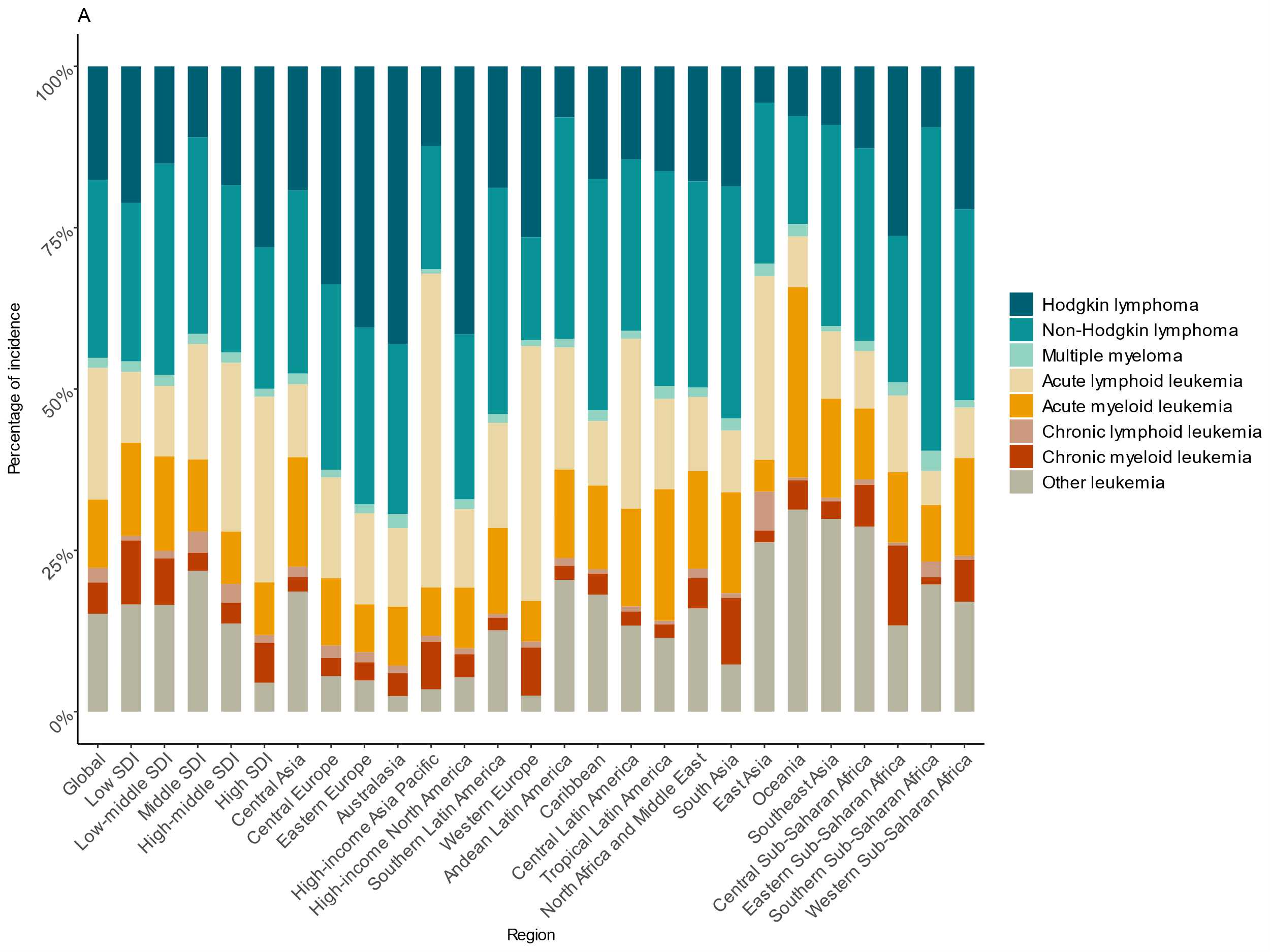


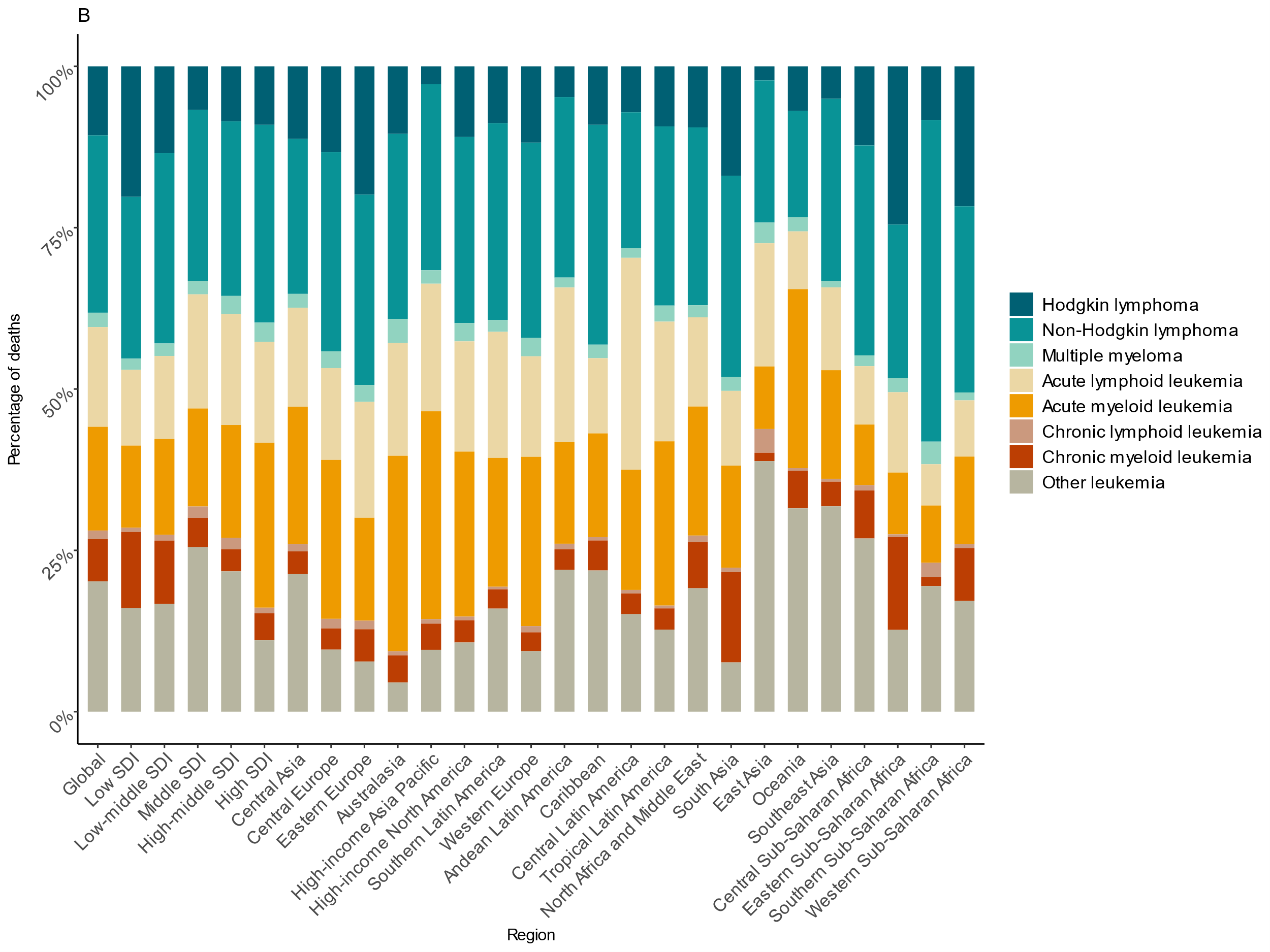


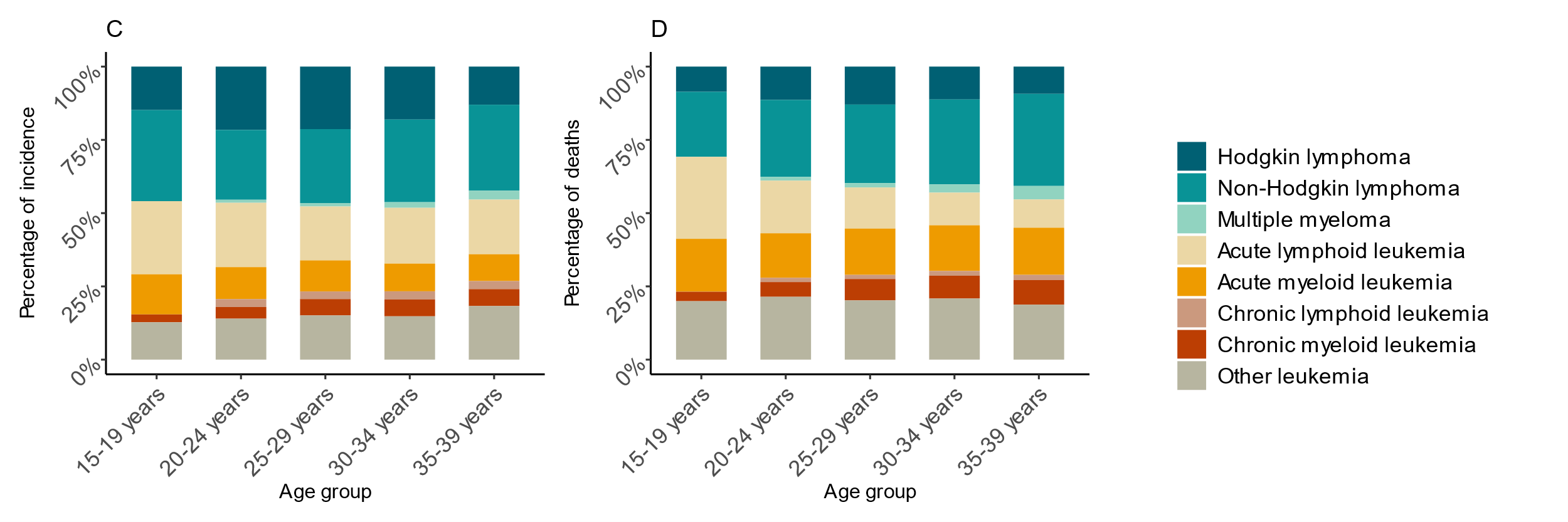


Figure S1 Proportional burden of incidence (A) and deaths (B) for hematological malignancies in adolescents and young adults across various regions in 2019. Proportional burden of incidence (C) and deaths (D) for hematological malignancies in adolescents and young adults across various age groups in 2019.


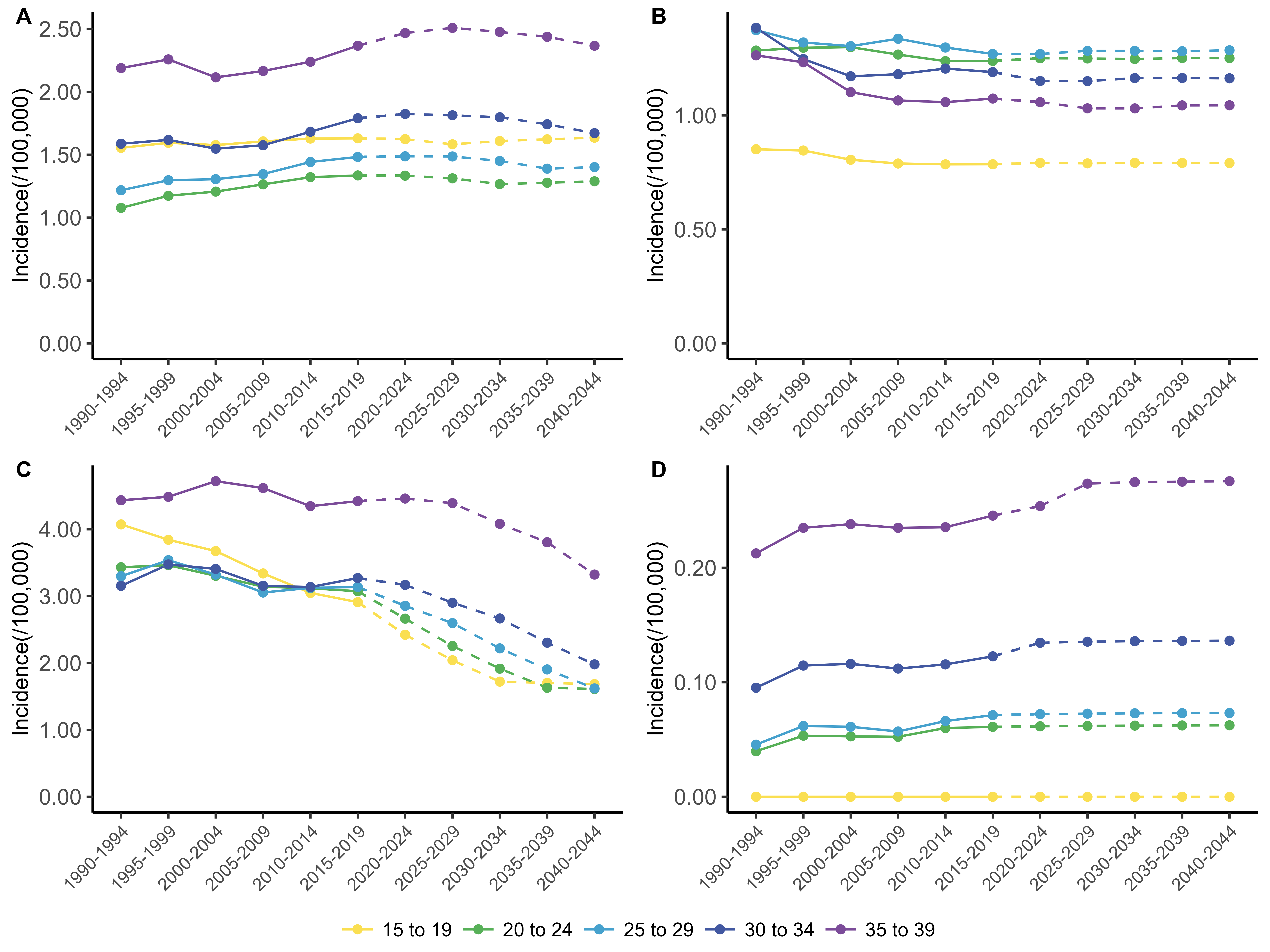


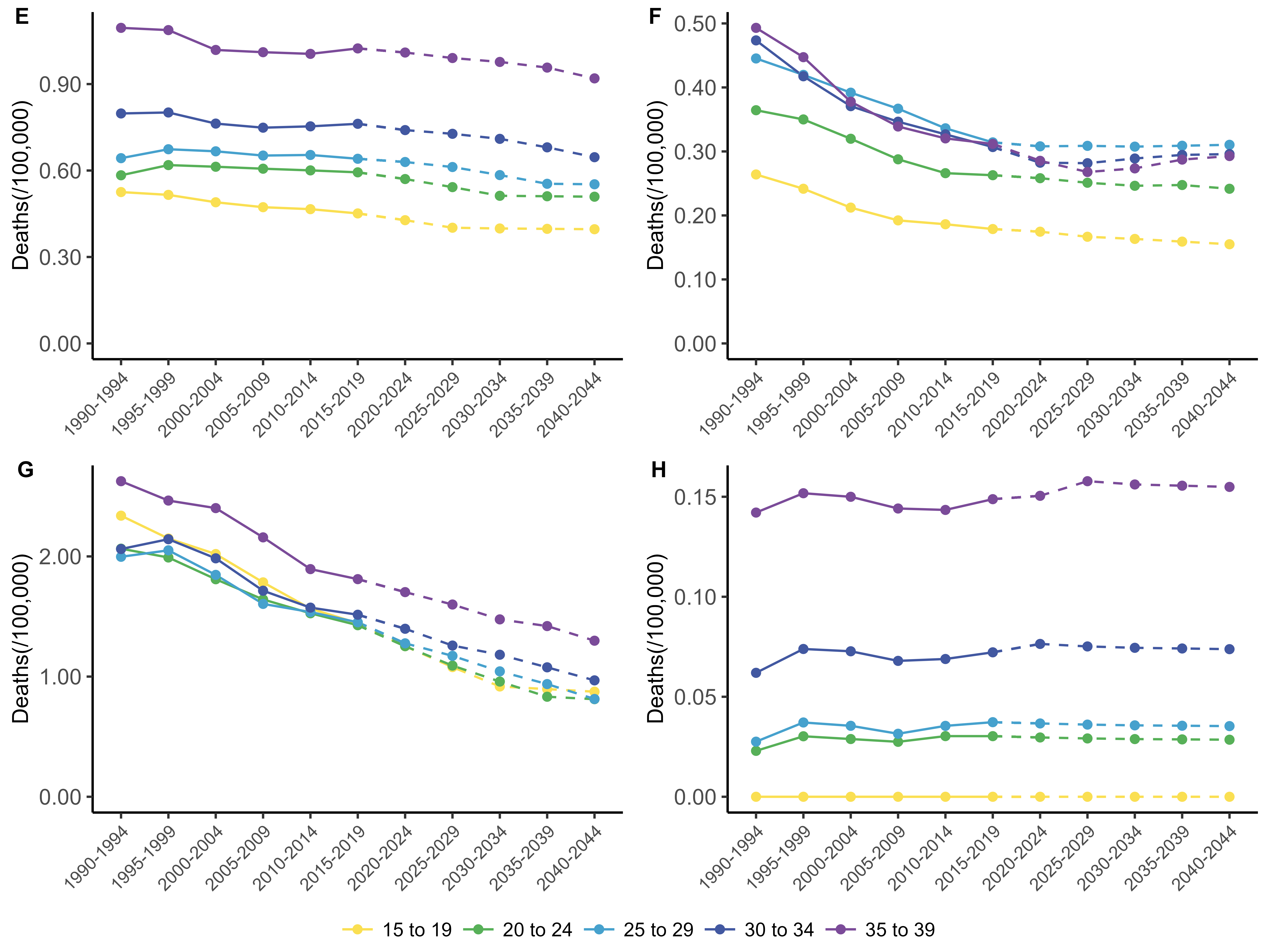


Figure S2 Trends in incidence rate of hematological malignancies for different age group, from 1990 to 2044. A: non-Hodgkin's lymphoma; B: Hodgkin's lymphoma; C: Leukemia; D: Multiple myeloma. Trends in mortality rate of hematological malignancies for different age group, from 1990 to 2044. E: non-Hodgkin's lymphoma; F: Hodgkin's lymphoma; G: Leukemia; H: Multiple myeloma.
